# Supplementary material for: Women’s experiences of over-the-counter and prescription medication during pregnancy in the UK: findings from survey free-text responses and narrative interviews
Source: BMJ Open. 2023 Feb 20;13(3):e067987. doi: 10.1136/bmjopen-2022-067987 (PMC9990671; doi:10.1136/bmjopen-2022-067987)
Supplement: Supplementary data [file bmjopen-2022-067987supp001.pdf]

# WRISK Topic Guide

## Consent

Thank you … I'm part of a small research team run by the British Pregnancy Advisory Service and researchers at Cardiff University. Today I'd like to explore your thoughts, opinions, and experiences of information and advice you received about your last pregnancy.

**Check:** are you still happy to be involved with the research?

Just to check, I will be recording the interview… **(If face-to-face, show participant the audio-recorder).** It will help me to remember what we talk about and means I do not have to make loads of notes whilst we are talking. The tape will not be played to anyone outside of the research team.

Are you happy with that?

**Read written consent form with participant, checking;**

- **understand the statement,**
- **agree with it, and,**
- **have had opportunity to ask questions and that boxes are all checked.**

Our conversation will remain confidential.
You will not be named in anything I write about this research.

If there is anything you don't want to talk about just say so- some questions may involve recalling distressing experiences. This interview is about your experiences. If we start to discuss something that you would rather not, please let me know and we can talk about something else.

These interviews usually last about one hour, but we can stop whenever you like. If you would like to take a break, please just let me know.

## About you and your most recent pregnancy

Perhaps we can start with you and your family … can you tell me a little bit about that …

**Allow participant to talk, In course of which ask…**

- **Number and ages of any children …**
- **Most recent pregnancy…**
- **Other pregnancies, miscarriages, terminations …**
  **(but take a judgement about not interrupting flow)**

Okay, so thinking a little bit about your most recent pregnancy ….
Can you tell me a little about that?

- What would you say were the highpoints- Why?
- What would you say were the low points - Why?



Sanders J, *et al. BMJ Open* 2023; 13:e067987. doi: 10.1136/bmjopen-2022-067987

## Remaining topics can be covered in any order.

### In wider life context

Women often have a lot going on in their lives during the time when they are pregnant … for some people it can be a time of when everything seems to happen at once…

What else was going on for you at that time?

**Explore – relationships and partners, housing, work, education …**

### Becoming pregnant

So… would you say this pregnancy was …

- Planned, or sort-of planned, or a surprise …

Thinking back to the months before became pregnant …
Did you have any concerns about…

- **Living circumstances, work, family life**
- **Fertility or about being pregnant**
- **Your own health**

Thinking back, do you remember getting any information or advice about looking after yourself before becoming pregnant? What? And Who?

- **Health professionals**
- **Books / social media**
- **Friends and family**

Did you seek out this advice yourself?

Did you get any help that you felt was particularly helpful? Unhelpful?

Was there anything you wish someone had told you then?

### Finding out

Now, thinking back to when you found out that you were pregnant…
Can you tell me a little bit about that?

**Prompt**

- **How did you feel about finding out that you were pregnant?**
- **Did you have any particular concerns?**
  - **Health**
  - **Work, housing, social life**
  - **Life circumstances/partners/support**



Sanders J, *et al. BMJ Open* 2023; 13:e067987. doi: 10.1136/bmjopen-2022-067987

Did anyone advise or tell you anything that you felt was particularly helpful / unhelpful at that time? Is there anything you would like to have known?

What? And Who?

Did you look for any information/advice yourself? For example on the internet, in books, or magazines?

## Stories in the media

There are often lots of stories about health and pregnancy on social media, newspapers, magazines, tv and online, talking about things mothers should and shouldn't do to conceive or have a healthy pregnancy and baby. Have you been aware of any stories like that?

**Prompt – for example, about what to eat or drink, or activities not to take part in?**
**Prompt – what did you think about those stories?**
**Prompt- how did they make you feel, if anything?**
**Prompt – did any stories feel particularly relevant to you?**
**Prompt – did you make any changes because of the stories you had read?**

## Health and medications

Many women take medication while they are pregnant – did you take any medications?

**If yes… which ones? When?**

Did you get any information or advice from health professionals, friends, family members or from social and mainstream media about taking medication?

**If yes… explore, If no… why not… explore**

How did you feel about the advice or information that you got?

Did you feel that advice from different people was broadly similar or not?
Was there anything that you felt was particularly helpful? / Particularly unhelpful?
Did you decide to do anything differently?

## Lifestyle advice

Sometimes women are given specific information about things like eating and drinking in pregnancy from health professionals or family members or friends or from social media or mainstream media … do you remember receiving any information or advice about…

- **Healthy lifestyle**
- **Weight and exercise**
- **Alcohol**
- **Smoking**
- **Cannabis, Cocaine, Ecstasy**

What advice/ information? And who gave it?

Was there anything that you felt was particularly helpful? / Particularly unhelpful?



Sanders J, *et al. BMJ Open* 2023; 13:e067987. doi: 10.1136/bmjopen-2022-067987

Did you decide to do anything differently? Why? Did you change the way you did things because you wanted to or because you felt under pressure to?

## Weight and fitness

Sometimes women are given information or advice about managing their weight and general fitness.

Did you get any advice and information or information about managing your weight or level of fitness?
**If yes… explore,**

Can you tell me about any information or advice you were given?
How did you feel about that advice?

Was there anything that you felt was particularly helpful? / Particularly unhelpful?
Did you decide to do anything differently?

## Antenatal appointments and scans

I would like to hear about your experience of going for antenatal care and scans…

Can you tell me about your experience of going for a scan?
- What info or advice did you have? Helpful? Not?

What advice did you receive about antenatal care? And who gave it? (Probe third sector orgs etc. if appropriate)

Was there anything that you felt was particularly helpful? / Particularly unhelpful?

Did you decide to do anything differently?

## Planning for birth

Thinking now about the birth of your baby…. Can you tell me a little about that?

Did things go the way you hoped they would?

Explore how decisions were made and how mother felt about those decisions… including exploring any sources of risk advice – health professional (specifically which one?), friend or family member, social media, antenatal teacher etc… associated with ….

- **Type of delivery**
- **Assisted?**
- **Place of birth**
- **Timing of delivery/induction**
- **Probe: quantitative presentation of risk information**

Do you think the information you were given helped you make the decision that was right for you?
Did you feel listened to and respected?

## Looking back

Looking back along the whole of your pregnancy, if you could go back in time and do something differently in the time before you became pregnant … what would that be? Why?

**[IF APPLICABLE: Anything different compared to previous pregnancies?]**

And again, thinking back, is there anything you wished you had or hadn't known?

If you could change anything about the advice and support that you received during pregnancy and birth, what would it be …

## Final thoughts and thank you

We have almost come to the end of our time together. Is there anything else you would like to add to our conversation today?

Thank you so much for your time today. Everything you have shared today has been so helpful.

**Thank participant and give (or remind that you are emailing) a £20 gift voucher as a thank you.**

I'm going to turn off the recorder now.

I am going to go away and transcribe what I have recorded today. You can contact me at any time to discuss what we have shared or if you have any questions about the research.



Sanders J, *et al. BMJ Open* 2023; 13:e067987. doi: 10.1136/bmjopen-2022-067987
