# Supplementary material for: Women’s experiences of over-the-counter and prescription medication during pregnancy in the UK: findings from survey free-text responses and narrative interviews
Source: BMJ Open. 2023 Feb 20;13(3):e067987. doi: 10.1136/bmjopen-2022-067987 (PMC9990671; doi:10.1136/bmjopen-2022-067987)
Supplement: Author's manuscript [file bmjopen-2022-067987.draft_revisions.pdf]

BMJ Open

BMJ Open is committed to open peer review. As part of this commitment we make the peer review history of every article we publish publicly available.

When an article is published we post the peer reviewers' comments and the authors' responses online. We also post the versions of the paper that were used during peer review. These are the versions that the peer review comments apply to.

The versions of the paper that follow are the versions that were submitted during the peer review process. They are not the versions of record or the final published versions. They should not be cited or distributed as the published version of this manuscript.

BMJ Open is an open access journal and the full, final, typeset and author-corrected version of record of the manuscript is available on our site with no access controls, subscription charges or pay-per-view fees (http://bmjopen.bmj.com).

If you have any questions on BMJ Open's open peer review process please email info.bmjopen@bmj.com

BMJ Open

‘Try not to take them’: Women’s experiences of over the counter and prescription medication during pregnancy in the UK

Journal:	*BMJ Open*
Manuscript ID	bmjopen-2022-067987
Article Type:	Original research
Date Submitted by the Author:	05-Sep-2022

Complete List of Authors:	Sanders, Julia; Cardiff University, School of Healthcare Sciences<br>Blaylock, Rebecca; British Pregnancy Advisory Service, Centre for Reproductive Research & Communication<br>Dean, Caitlin; Pregnancy Sickness Support<br>Petersen, Irene; University College London Medical School, Department of Primary Care and Population health<br>Trickey, Heather; University of Cardiff, Social Medicine<br>Murphy, Clare; British Pregnancy Advisory Service, Centre for Reproductive Research & Communication

Keywords:	CLINICAL PHARMACOLOGY, Reproductive medicine < GYNAECOLOGY, Maternal medicine < OBSTETRICS, PUBLIC HEALTH, QUALITATIVE RESEARCH

SCHOLARONE™
Manuscripts

I, the Submitting Author has the right to grant and does grant on behalf of all authors of the Work (as defined in the below author licence), an exclusive licence and/or a non-exclusive licence for contributions from authors who are: i) UK Crown employees; ii) where BMJ has agreed a CC-BY licence shall apply, and/or iii) in accordance with the terms applicable for US Federal Government officers or employees acting as part of their official duties; on a worldwide, perpetual, irrevocable, royalty-free basis to BMJ Publishing Group Ltd ("BMJ") its licensees and where the relevant Journal is co-owned by BMJ to the co-owners of the Journal, to publish the Work in this journal and any other BMJ products and to exploit all rights, as set out in our licence.

The Submitting Author accepts and understands that any supply made under these terms is made by BMJ to the Submitting Author unless you are acting as an employee on behalf of your employer or a postgraduate student of an affiliated institution which is paying any applicable article publishing charge ("APC") for Open Access articles. Where the Submitting Author wishes to make the Work available on an Open Access basis (and intends to pay the relevant APC), the terms of reuse of such Open Access shall be governed by a Creative Commons licence – details of these licences and which Creative Commons licence will apply to this Work are set out in our licence referred to above.

Other than as permitted in any relevant BMJ Author's Self Archiving Policies, I confirm this Work has not been accepted for publication elsewhere, is not being considered for publication elsewhere and does not duplicate material already published. I confirm all authors consent to publication of this Work and authorise the granting of this licence.

For peer review only

Title

'Try not to take them': Women's experiences of over the counter and prescription medication during pregnancy

Julia Sanders[a]
Rebecca Blaylock[b]
Caitlin Dean[c]
Irene Petersen[d]
Heather Trickey[e]
Clare Murphy[b]

[a] School of Healthcare Sciences, Cardiff University, United Kingdom
[b] Centre for Reproductive Research & Communication, British Pregnancy Advisory Service, 30-31 Furnival Street, London, United Kingdom
[c] Pregnancy Sickness Support, 19G Normandy Way, Bodmin, United Kingdom
[d] Department of Primary Care & Population Health, University College London, United Kingdom
[e] School of Social Sciences, Cardiff University, United Kingdom

Emails and ORCID IDs

Julia Sanders sandersj3@cardiff.ac.uk 0000-0001-5712-9989
Rebecca Blaylock rebecca.blaylock@bpas.org 0000-0003-4317-1638
Caitlin Dean caitlin@pregnancysicknesssupport.org.uk 0000-0002-8812-5101
Irene Petersen i.petersen@ucl.ac.uk 0000-0003-2446-0506
Clare Murphy clare.murphy@bpas.org 0000-0002-7272-9605

Corresponding author

Rebecca Blaylock
Research & Engagement Lead
Centre for Reproductive Research & Communication
British Pregnancy Advisory Service
30-31 Furnival Street
London
EC4A 1JQ
Email: rebecca.blaylock@bpas.org

For peer review only

Abstract

Objectives: To explore women's experiences of over the counter and prescription medication advice and use during pregnancy.

Design: A study design consisting of an online survey and nested in-depth interviews with a sub-sample of participants.

Setting: The United Kingdom.

Participants: A total of 7090 women completed the survey, 34 women who collectively had experienced 68 pregnancies, were subsequently interviewed. Women were eligible if living in the UK, aged 16-45 years, were pregnant or had been pregnant in the last five years regardless of pregnancy outcome.

Results: Medication prescribing and use during pregnancy was common and some medications were prescribed and taken without apparent question or concern. The prescribing, dispensing, and taking of some advised medications was restricted through women's or prescribers' fear of fetal harm. Lack of adherence to national prescribing guidance, conflicting professional opinion, and poor communication resulted in maternal anxiety, avoidable morbidity, and women negotiating complex and distressing pathways to obtain recommended medications.

Conclusions: Increased translation of national guidance into practice, and greater personalisation of antenatal care are needed to improve the safety, efficacy, and personalisation of prescribing in pregnancy.

Strengths and limitations of this study

- The study gained new insights into a seldom researched topic
- A sampling frame ensured inclusion of women with social and medical complexities from across the UK
- Women without sufficient spoken English or internet access were excluded
- Interviews were conducted only with women, not the health professionals who prescribe

For peer review only

Title

'Try not to take them': Women's experiences of over the counter and prescription medication during pregnancy in the UK

Background

Safe and effective prescribing is an essential component of antenatal care. Prescribing in pregnancy requires additional knowledge and caution due to the potential for teratogenesis, altered pharmacokinetics, maternal concerns[1], and potential for short and longer term harm to the baby[2 3]. Antenatal information[4] provided to pregnant women states that most medications reach the unborn baby and all medication use should be discussed with health professionals. Many women discontinue or avoid medications in pregnancy[5], however, lack of treatment can potentially have severe consequences[1]. Despite selective avoidance, medication use in pregnancy is common with around 87% of pregnant women in the UK reporting medication use for short term or chronic conditions[6]. Excluding vitamin and iron supplements, frequently prescribed and over the counter (OTC) medications taken during pregnancy include antacids, analgesics, anti-emetics, and antibiotics[6].

There are few medications that should not be used by pregnant women for example thalidomide, sodium valproate and isotretinoin due to their teratogenicity. Most other medications are safe and widely used during pregnancy and there are several National prescribing guidelines[7 8] for treatment during pregnancy. For example, the National Institute for Health and Care Excellence (NICE) guidance on antenatal and postnatal mental

health recommend that health professionals should discuss the potential benefits of psychological interventions and psychotropic medication, the possible consequences of no treatment and possible harms associated with treatment and what might happen if treatment is changed or stopped, particularly if psychotropic medication is stopped abruptly[8]. For mild to moderate mental health problems psychological interventions should form first line management[8], but access is limited by long waiting lists and high referral thresholds, whilst a lack of continuity in antenatal care leads to difficulty developing trusting therapeutic relationships[9]. Suicide remains an important avoidable cause of pregnancy-associated death[10], and reports have highlighted that treatments for depression may often be discontinued in pregnancy[5 10].

The Royal College of Obstetricians and Gynaecologists have set out clear guidance on the diagnosis and subsequent management of nausea and vomiting in pregnancy across community, ambulatory day-care, and inpatient settings, including a medication escalation ladder for use when first line recommended treatments are ineffective[7].

There is growing concern that public health messages aimed at pregnant women, including those relating to medication use, do not always fully reflect or explain the evidence base underpinning them and the nuances and complexity of information is lost[11]. Thus, the inability to receive effective medications is not without consequence for women and babies. A survey in 2015 identified women with severe hyperemesis gravidarum frequently had difficulty obtaining swift treatment and support for debilitating pregnancy sickness resulting in some terminating an otherwise wanted pregnancy[12]. Likewise, although mental health problems are common during pregnancy[13] previous reports have highlighted barriers to access to appropriate treatment[14].

The 'WRISK project: Understanding and improving the way risk in pregnancy is communicated to women' was established to explore women's experiences of maternity related public health and risk messaging, including those relating to medication use in pregnancy. The study aimed to hear women's voices through public involvement, quantitative, and qualitative methodologies with a specific objective of exploring the views of women previously identified as feeling stigmatised or poorly served by current practice[15] .

For peer review only

This paper focuses specifically on women's experiences of medication use in pregnancy as reported in open text comments in the survey and in-depth interviews.

Methods

Study design

The study design consisting of an online survey and nested in-depth interviews with a sub-sample of survey participants. The survey was open to UK residents aged 16-45 years, who were pregnant or who had been pregnant in the last five years, regardless of pregnancy outcome. The survey was publicised through social media platforms including those of the study's charity partners. We invited survey respondents to express interest in further research involvement and recruited participants for the interviews from this population. As the survey was exploratory, an a priori sample size calculation was not required.

For subsequent in-depth interviews we used a sampling frame to ensure we heard the voices of women at greater risk of the most severe pregnancy outcomes, maternal death or stillbirth[10 16]. The frame aimed to ensure the woman interviewed included a minimum of 20% who were eligible for means-tested state benefits, a minimum of 20% from Black, Asian, and Minority Ethnic backgrounds, and at least six women were interviewed with experience of the following pregnancy experiences: pregnancy <20 years of age, Body Mass Index >30Kg/m^2, antenatal mental health problems or experience of hyperemesis gravidarum. Interested respondents who met the sampling criteria were selected using a random number table, until required numbers were obtained.

Data Collection

Survey

A participant information sheet and consent form were integrated into the survey. The survey included questions on participants' experience of the advice, information, and support they received from different sources during pregnancy, with the focus on the respondent's most recent pregnancy. Questions with open free-text responses were included alongside questions with Likert scales and multiple-choice responses. We describe the survey and demographic characteristics of responders in full in a previous publication[15].

For the purposes of this publication, we present sociodemographic data and free text responses from the survey pertaining to medication use in pregnancy only.

One-to-one interviews

The participant information sheets, consent forms, and topic guides for the interviews were developed in collaboration with the combined researcher and charity project advisory group. Narrative topic guides allowed participants to tell their pregnancy stories and were tailored to various pregnancy outcomes. Participants were asked to primarily discuss their most recent pregnancy and were asked about antenatal prescribing and use of over-the-counter medications, amongst other topics.

Participants were offered the option of in-person or telephone interviews. Interviewers [RB and HT] made detailed field notes following each interview. Participants were offered, and all received a high street vouchers worth £20. Audio files were transcribed verbatim using a commercial transcription service. Electronic transcripts were stored separately to any identifiable data on a secure IT system, and audio files deleted once they had been checked for accuracy.

Research team

The trained, all female research team had extensive experience of working in pregnancy-related research and practice including social sciences (RB and HT), medical law (JS), public health (RB, JS, and HT) and clinical midwifery (JS). Interviews were conducted and analysed by RB and HT.

Terminology

The WRISK project was inclusive of all people who had experienced pregnancy in the previous five years. The project team always referred to individuals according to their self-determined gender. In this paper we use the words ‘woman/ women’ as the vast majority of participants self-identified as women. We use the term “BAME”, but acknowledge that this is problematic[17].

For peer review only

Data analysis

The interviews and free text comments in the survey were analysed thematically following Braun and Clarke's method[18]. Transcripts were coded and analysed using Dedoose[19]. All transcripts and accompanying data such as researcher's field notes were read in detail several times by both interviewers, and high-level codes pertaining to the original research questions were identified. Further codes were identified in the data during an inductive process, resulting in a coding framework informed by the data itself. Narrative summaries of data pertaining to each code were produced and cross-checked by RB and HT, and subsequently organised into high-level themes. We used Excel® and STATA® to generate descriptive statistics on the survey and interview populations[20 21].

Patient and Public Involvement

The project oversight group included representatives from five diverse maternity user groups, healthcare professionals, and researchers in addition to the study team. The group met regularly throughout the project from conception to dissemination and collectively informed all aspects of study design and delivery.

Results

The survey was completed by 7090 women, of whom 3,175 (44.1%) expressed willingness for further involvement, and 34 were subsequently interviewed. Interviews were carried out by RB and HT between July and November of 2019, lasting approximately 45-60 minutes. Two pilot interviews were conducted in-person in April 2019 and were deemed to be of sufficient quality to be included in the analysis. All subsequent interviews were conducted by telephone and recorded using a Dictaphone. The 34 women interviewed included those with experience of: a BMI >30, (n=7); antenatal medication use for mental health conditions, (n=6); medication for hyperemesis gravidarum, (n=9); age <20 during pregnancy, (n=7); or having a termination due to a perceived or actual risk either to themselves or their baby (n=7). Some participants fitted more than one category, whilst five had none of these experiences. Sociodemographic characteristics of those interviewed are described in Table 1 and sociodemographic characteristics of all survey respondents are published elsewhere[15].

For peer review only

Table 1

Sociodemographic characteristics of interview participants at time of interview

	n (34)	%		Mean	Range
**SOCIODEMOGRAPHICS**					
Age					
**16-18**	0	0		-	-
**19-20**	2	5.9		-	-
**21-25**	9	26.5		-	-
**26-30**	7	20.6		-	-
**31-35**	7	20.6		-	-
**36-40**	7	20.6		-	-
**41-45**	0	0		-	-
**45+**	1	2.9		-	-
**Missing**	1	2.9		-	-
Highest level of education					
**Secondary school**	2	5.9		-	-
**Apprenticeship/HND/NVQ**	3	8.8		-	-
**A-Levels**	6	17.6		-	-
**Undergraduate degree**	13	38.2		-	-
**Postgraduate degree**	10	29.4		-	-
**Missing**	0	0		-	-
Relationship status					
**Married**	23	67.6			
**Have a partner & live with them**	9	26.5			
**Have a partner & live separately**	1	2.9			
**Polyamorous**	1	2.9			
Ethnicity					
**White: English/Welsh/Scottish/Northern Irish/British**	24	70.6			
**Black/African/Caribbean/ Black British: African**	3	8.8			
**Mixed/multiple ethnic groups: White & Black African**	1	2.9			
**Asian/Asian British: Chinese**	1	2.9			
**Asian/Asian British: Indian**	1	2.9			
**Black/African/Caribbean/ Black British: Caribbean**	1	2.9			

For peer review only

**Mixed/multiple ethnic groups: White & Black Caribbean**	2	5.9			
**Mixed/multiple ethnic groups: White British & Middle Eastern**	1	2.9			
Gender					
Female	33	97.1			
Non-binary	1	2.9			
Receive State benefits?					
**Yes**	10	29.4			
**No**	23	67.6			
**Missing**	1	2.9			
**PREGNANCY HISTORY**					
Gravidity	-	-		2	1 – 5
Live births	-	-		1.2	1 – 4
Terminations/abortion	-	-		0.3	0 – 2
Miscarriage/stillbirth	-	-		0.3	0 – 2

Four themes were identified in relation to medication use in pregnancy: 'fear of medications and self-regulation', 'feeling overmedicated', 'conflicting opinions', and 'running the gauntlet'. Some themes related to women's experiences of medication use in general, while others were condition specific.

Fear of medications and self-regulation

Fear, or anxiety for the potential for fetal harm caused by over the counter and prescribed medications were expressed by women. They also reported prescribers' reluctance to prescribe for this reason. Participants expressed support for the precautionary approach that medications should be kept to a minimum during pregnancy. The fear of causing fetal harm through taking medication resulted in women reducing medication even when this was required to control serious medical conditions.

Two women described the consequences of stopping prescribed medication for pre-existing medical conditions. One woman stopped taking her asthma medication which resulted in hospitalisation:

> *"I stopped taking my inhaler, just because I just didn't want to harm the baby. I took a bad cold, and I was coughing up blood, and then they sent me to hospital for all these other tests. They thought I had a clot or*

For peer review only

something. So that obviously scared me. Then I just said, Screw it. I'm just going to take my asthma medication, and that was that." [WRI28]

Similarly, another woman experienced a serious migraine and temporary blindness, following discontinuation of medication for cranial hypertension:

"I have a brain condition called... Well, it's cranial hypertension, so it just means that my body produces too much spinal fluid, which pushes on my brain, which causes migraines, essentially. … because there isn't a lot of research into this condition in pregnancy, I decided, personally, to stop taking the medication. Yes, so I stopped all my medication and, thankfully, touch wood, everything was fine. … I did struggle with a few migraines throughout the pregnancy, one which was quite severe, which resulted in a loss of vision in my left eye, but luckily, it came back. So that was fine. That was quite difficult to deal with, though." [WRI23]

Self-regulation of medication particularly related to the use of analgesics. Women described conflict between their understanding of some medications being safe to take in pregnancy, with their desire to try "not to take them" [WRI11] due to the possibility of potential risks to the fetus.

"… we all know it's [paracetamol] is generally safe, it's one of the safest painkillers there is, so it's the only one you can take, but even just taking that, women don't like- you know, they have a headache instead of taking the medicine, because they feel so guilty." [WRI30]

Another woman described managing with paracetamol for pelvic girdle pain even after she had been prescribed codeine-based medication:

"Yes, one of those [codeine-based analgesia] and I was reading the side effects and I was like, 'No, I'm not happy taking it and having that go into my baby.' I just literally was taking paracetamol and I was just trying to take the lowest dose of paracetamol that I could really." [WRI4]

For peer review only

Whilst women generally tried to avoid prescribed medication, recommended vaccines, including for influenza, appeared to be accepted. Supplements were viewed as positive and often taken without any apparent fear of causing an adverse pregnancy outcome.

"Interviewer: Did you take medications while you were pregnant?

No, not at all.

Interviewer: No, not at all. Okay.

Beyond supplements and the flu jab and recommended stuff like that. I had a whooping cough jab, I think, with [daughter's name] but no regular medication of any sort." (WRI7)

There were many examples of health professionals reinforcing the message that medication should be avoided in pregnancy.

"Throughout my pregnancy I really struggled with my mental health. So, at one point, I don't know how pregnant I was, I must have been about six or seven months, we got an emergency appointment at my GP's surgery… and we said, 'I'm really struggling. Is there anything you can do?' His first reaction was that I shouldn't be on any medication because I was pregnant." [WRI29]

Health professions expressing their own concerns around prescribing had a particularly negative impact on women. One interviewee [WRI17] explained her concern when a hospital consultant prescribing "put his hands together in prayer and said, '*God, forgive me for giving you this. I hope your baby's okay'*" when prescribing ondansetron for severe hyperemesis gravidarum (HG).

Other women had similar experiences when accessing medication for their mental health. WRI33 said:

"because I was being so sick, I went to the doctor's about anti-sickness medication. She saw I was on antidepressants and she made me feel like I was the world's worst mother. I don't know whether she meant to, but she was just worried that, 'Ooh, you're pregnant and you're on medication.' She made me feel like, 'You're hurting your baby'. Then when I went to the pharmacy to pick up my meds, they made me feel like it on

For peer review only

the same day as well. I think that was the point where I started to really decline in my mental health. It just seemed to trigger something off that I was going to be a terrible mother."

Feeling overmedicated

Some participants reported feeling overmedicated, and that pharmacological treatments were used without exploring other options first. This was the case for women with both mental and physical health concerns:

"I feel like they push drugs on people too quickly, that is just the go-to. I had a traumatic birth with number two. That's why I went on the antidepressants with number three, because I was terrified of giving birth. They just kept telling me baby was getting so big, blah blah blah. I was, literally, terrified of giving birth to her. They just put my antidepressants. There was no, 'Let's talk about it, get counselling, and get over the traumatic birth.' It was just mask it really. I don't think that's right to do when you're dealing with, obviously, a pregnant woman, a baby. [WRI31]

Current guidance relating to gestational diabetes recommends metformin is introduced if good blood glucose control is not achieved with 1 – 2 weeks of dietary change and exercise[22]. One survey respondent would have preferred to extend this duration before commencing medication.

"I would have liked to have been better involved in decision making around my options for treatment in relation to my gestational diabetes... I ended up with 2 tablets of metformin which I think was overkill - I would have liked to have had the opportunity to try harder with diet and exercise before starting medication."

Conflicting opinions

Women who required prescribed medication in pregnancy described frustration and distress resulting from conflicting opinions of health professionals. This conflict was particularly felt

For peer review only

by women with hyperemesis gravidarum or mental health problems. One woman who required ondansetron for severe hyperemesis gravidarum was prescribed it, later to have this decision questioned by another General Practitioner [GP] in the same practice:

"So, I was prescribed by one doctor the ondansetron and because I was running out, they said I had to speak to another doctor just to check it was obviously working, just a check-up on it, to get the repeat prescription. Then when this doctor phoned me, she said, 'You shouldn't be taking this. This is for cancer patients. There's no reason why you should be taking it. You're putting your baby's life at risk'. … She just wasn't having any of it. I even told her that I'd just had a scan like 45 minutes before the phone call, and she said, 'Oh well, that can change at your 20 week scan. Don't come crying to me when they tell you that your baby has got heart defects'". [WRI15]

Another participant described the distress that resulted when a pharmacist would not fulfil a prescription for anti-depressants prescribed by her GP.

"They just wouldn't give me my prescription, and people were behind me in the queue and could hear what I was on, that I was pregnant. I wasn't 12 weeks yet, so I hadn't told anybody. I had to just sit there waiting for them to decide whether I could have it or not and then they said, 'No, you can't have it.' I left without any medication, and I was just crying in the car." [WRI33]

Two interview participants said that conflicting and contradictory advice from healthcare professionals was "staggering".

"I found through everything in my pregnancy that all the doctors I saw had completely different advice and completely different opinions to each other..." [WRI6]

For peer review only

The conflict was seen across all professional groups. One respondent described conflicting information from her midwife and GP.

"I was advised by my GP to continue my anti-depressant medication. My midwife continually questioned this decision throughout the whole pregnancy". [Survey respondent]

Running the Gauntlet

This theme particularly related to prescribing for hyperemesis gravidarum. Some women experienced prolonged periods prior to getting effective treatment. Four women who were suffering with hyperemesis gravidarum described their experience of the current guidance.

"they tried me on cyclizine, which just made me dizzy and did nothing for the sickness. I went very dizzy and sleepy. And then Stemetil… Which also had no effect. So, I was, kind of, getting desperate. And then, … they gave me ondansetron which is what they give to chemo patients, you probably know, and it was like I was alive again. … They were sympathetic, but I didn't get a lot of advice on what effect it would have on the baby. Just, you know, like, with the Cyclzine and the Stemetil, they were very much, like, 'It's been used a lot and we can be certain from observations that it won't have any effect.' So that was fine. They were, kind of, reluctant to, like, move me onto the stronger stuff." ***[WRI1]***

"… they could see that I couldn't even take a sip of water without being sick, so I needed something. So, first off, I was given a tablet that you put between your gum and your teeth. It wasn't working. So, I went back and I got something called cyclizine, which is a tablet. That started to make it a little bit better for a couple of weeks and then I just got worse again. So, I had to go back again and …[they] gave me a pill called ondansetron. Then that was working, but I was still quite bad. So, I went back again and they gave me a combination of cyclizine and ondansetron, and that did the trick in the end". WRI6

For peer review only

Even when women were prescribed treatment for hyperemesis, they could then be discouraged from taking it by the prescriber:

> *"The most unhelpful piece of advice I received was from a doctor, who prescribed me anti-sickness tablets for hyperemesis but said 'try not to take them unless you feel you need them' because 'we can't say that any medicine is safe in pregnancy'. I was very unwell, very dehydrated and very disoriented and was afraid of causing harm to the baby so I didn't take them." [Survey respondent]*

Another woman who was prescribed medication received comments from a pharmacist who suggested they may be unnecessary:

> *"My husband went to collect my prescription when I was discharged, and was told that I was being 'irresponsible' and 'putting the baby at risk' by taking the medication. Later in pregnancy (I had HG until the moment of delivery), another pharmacist told me that I shouldn't still be taking medication as morning sickness would've stopped by my late stage, implied it was all in my head, and suggested that I try a herbal remedy instead." [Survey respondent]*

For peer review only

Discussion

This study adds important understanding to the seldom explored topic of women's experiences of antenatal medication use. Many women wished to reduce exposure to over the counter and prescribed medication use during pregnancy, but dietary supplements and vaccines, were generally accepted by pregnant women. This contrasted to treatments for mental health problems and hyperemesis gravidarum, conditions where pregnant women described having to negotiate conflicting opinions of health professionals to obtain recommended or effective treatments. Too often prescribing was more restrictive than recommended in national guidance resulting in avoidable, or prolonged, maternal morbidity, distress, and anxiety. Similar to the lack of preconceptual and antenatal care for women with epilepsy highlighted in reviews of maternal deaths[10], where information on medications used for chronic conditions was not shared, some women discontinued treatments without medical consultation, resulting in hospitalisation or exacerbation of symptoms.

In the UK, prescribing for pregnant women is undertaken by different health professionals which complicates communication. Women with existing medical conditions, including epilepsy, require preconceptual advice on medication use[23]. GPs are frequently involved in prescribing decisions, commonly prescribed medications such as iron therapy and low dose aspirin may be prescribed and administered by midwives. Women with additional obstetric needs will receive care led by obstetricians, who may share prescribing with GPs or specialist physicians. This arena is further complicated by public health bodies who are responsible for producing public health risk messages for pregnancy being independent from both primary and secondary care where prescribing occurs. This multidisciplinary approach to antenatal prescribing was found often to be fragmented, with women hearing conflicting opinions even from different members of the same professional group. The role of pharmacists as medication 'gatekeepers' and their refusal to dispense prescribed medications highlights the importance of their inclusion in system improvements.

Even when effective treatments were endorsed in national guidance, challenges in implementation were found. Despite RCOG guidance on the management of hyperemesis

For peer review only

gravidarum[7], women reported being denied access to effective treatments. Ensuring health professionals have easy access to up-to-date guidance on specialist aspects of care, and sufficient time and support to incorporate guidance into their practice is imperative. Whilst prescribers need to balance maternal benefit with potential fetal harm when prescribing in pregnancy, women's individual circumstances were not always considered, and they were not fully engaged in decision making. Possibly reflecting the tendency of health professionals to overestimate the teratogenic potential of drugs[24] we found many examples where health professionals used fear of fetal harm to justify a refusal to prescribe or dispense otherwise recommended medications. This had a significant impact not only on women's health, but on their emotional wellbeing with one reporting they were made to feel like the "world's worst mother".

Some women felt antidepressants and metformin for gestational diabetes were offered in preference to non-pharmacological options. This may reflect that the availability of talking therapies does not meet demand, or in relation to gestational diabetes a lack of informed personalised conversations on the effectiveness of methods to obtain glycaemic control.

High quality, easily accessible information on the safety of medicines is available[25] but appeared to be underutilised in informing individualised discussions. Better reporting systems on outcomes related to medication use in pregnancy are needed, including making the results of clinical trials more generalisable through, where appropriate, the inclusion of pregnant women[26]. Minimising prescribing in pregnancy became deeply entrenched in the ethos of antenatal care following the thalidomide tragedy[27]. The belief that all medication use during pregnancy carries risk is commonly held among women, with paracetamol, antibiotics and antidepressants[28] considered to be on a continuum of increasing risk. The potential for this position to cause harm is increasingly being realised. Some women with epilepsy, and others with serious mental health conditions have died because of an over stringent position on medication avoidance[10]. More recently the numbers of pregnant women who died during the covid-19 pandemic could have been reduced through earlier acceptance of the safety of vaccination in pregnancy, better public health communication, and greater use of effective treatments among those seriously ill with COVID-19[29]. While the need to reduce maternal mortalities through improved prescribing is already appreciated,

For peer review only

our study suggests physical and mental morbidity caused through the lack of access to effective treatments for mental health conditions and hyperemesis gravidarum is likely to be very common and requires improvement.

The strength of the study included adding to knowledge on a seldom explored topic, the geographical spread and high number of survey participants from across the UK and use of a sampling frame for the selection of interview participants. A weakness was that the sampling frame design may have shaped our findings and focused attention on certain experiences of medication use at the expense of others of equal concern to women. Our survey was self-selecting and may reflect the views of those more motivated to participate in research. The survey was only available in English and via the internet, excluding some groups from participation including non-English reading women, and those with limited or no access to the internet.

Conclusion

Medication prescribing and use during pregnancy is common and some medications were found to be prescribed and taken without question or concern. In contrast, fear of fetal harm restricts the prescribing and taking of some advised medications. Analgesics were commonly avoided or taken at lower than the therapeutic or prescribed dose and some women reduced or discontinued medications for chronic conditions without medical oversight. Where existing clinical guidance was not followed, or there was conflict in professional opinion, reluctance to prescribe or dispense resulted in women needing to negotiate complex and distressing pathways to obtain required medications. The study identified aspects of antenatal prescribing where improvement in knowledge, communication, or practice is required to ensure maximisation of the safety, efficacy, and personalisation of prescribing in pregnancy.

For peer review only

Funding statement

This work was supported by the Wellcome Trust as part of the WRISK Project [212089/Z/18/Z].

Data availability statement

Data are available on request.

Ethics statements

Patient consent for publication

Not applicable.

Ethical approval

Ethical approval was granted by the Research and Ethics committee of the School of Social Sciences at Cardiff University SREC/3201.

Word count

4,268

Author Contributions

CM and HT conceptualised the study and secured funding. CM, RB, HT, JS, IP and CD developed the protocol. HT and RB conducted the data collection and analysis. CM, JS, HT and RB provided study management. JS drafted the original manuscript. RB, IP and CD contributed to writing. CD and IP were members of the project advisory group. All authors approved the final manuscript.

Acknowledgements

We acknowledge and thank the group of over 7,000 women who shared their intimate stories of pregnancy with us. Special thanks go to the Oversight Committee: Dame Cathy Warwick, BPAS Chair of Board of Trustees and Chair of the Oversight Committee; Rebecca Brione, Birthrights; Jane Fisher, Antenatal Results and Choices; Caitlin Dean, Pregnancy Sickness Support; Elizabeth Duff, NCT; Professor Irene Petersen, University College London; Amber Marshall, BigBirthas.co.uk; Professor Fiona Woollard, Southampton University; Rolling representative, Public Health Wales.

For peer review only

We dedicate this paper to the late Dr Heather Trickey, without whom the project would never exist. Thank you for your inspiration, and dedication to the health and wellbeing of all women.

For peer review only

References

1. Twigg MJ, Lupattelli A, Nordeng H. Women's beliefs about medication use during their pregnancy: a UK perspective. *Int J Clin Pharm* 2016;38(4):968-76. doi: 10.1007/s11096-016-0322-5 [published Online First: 2016/05/30]
2. Meeraus WH, Petersen I, Gilbert R. Association between antibiotic prescribing in pregnancy and cerebral palsy or epilepsy in children born at term: a cohort study using the health improvement network. *PLoS ONE [Electronic Resource]* 2015;10(3):e0122034.
3. Page L. Prescribing for pregnancy: managing prescribing for women with mental health diagnoses. *Drug & Therapeutics Bulletin* 2020;58(1):8-11.
4. England N. Medicines in pregnancy 2022 [Available from: https://www.nhs.uk/pregnancy/keeping-well/medicines/ accessed 24 August 2022.
5. Petersen I, Gilbert RE, Evans SJ, et al. Pregnancy as a major determinant for discontinuation of antidepressants: an analysis of data from The Health Improvement Network. *J Clin Psychiatry* 2011;72(7):979-85. doi: 10.4088/JCP.10m06090blu [published Online First: 2011/04/05]
6. Lupattelli A, Spigset O, Twigg MJ, et al. Medication use in pregnancy: a cross-sectional, multinational web-based study. *BMJ Open* 2014;4(2):e004365. doi: 10.1136/bmjopen-2013-004365
7. Royal College of Obstetricians and Gynaecologists. The Management of Nausea and Vomiting of Pregnancy and Hyperemesis Gravidarum. Green-top Guideline No 69. London, 2016.
8. National Insitute for Health and Care Excellence. Antenatal and postnatal mental health: clinical management and service guidance. Clinical Guideline 192. 2020
9. Megnin-Viggars O, Symington I, Howard LM, et al. Experience of care for mental health problems in the antenatal or postnatal period for women in the UK: a systematic review and meta-synthesis of qualitative research. *Arch Womens Ment Health* 2015;18(6):745-59. doi: 10.1007/s00737-015-0548-6 [published Online First: 2015/07/18]
10. Knight M BK, Tuffnell D, Patel R, Shakespeare J, Kotnis R, Kenyon S, Kurinczuk JJ (Eds.) on behalf of MBRRACE-UK,. Saving Lives, Improving Mothers' Care - Lessons learned to inform maternity care from the UK and Ireland Confidential Enquiries into Maternal Deaths and Morbidity 2016-18. . Oxford: National Perinatal Epidemiology Unit, University of Oxford 2020.
11. Marshall O, Blaylock R, Murphy C, et al. Risk messages relating to fertility and pregnancy: a media content analysis. *Wellcome Open Res* 2021;6
12. Dean CMC. I could not survive another day. Improving treatment and tackling stigma: lessons from women's experience of abortion for severe pregnancy sickness: British Pregnancy Advisory Service, 2015.
13. Savory NA, Hannigan B, John RM, et al. Prevalence and predictors of poor mental health among pregnant women in Wales using a cross-sectional survey. *Midwifery* 2021;103:103103. doi: https://doi.org/10.1016/j.midw.2021.103103
14. Stevenson F, Hamilton S, Pinfold V, et al. Decisions about the use of psychotropic medication during pregnancy: a qualitative study. *BMJ Open* 2016;6(1):e010130. doi: 10.1136/bmjopen-2015-010130
15. Blaylock R, Trickey H, Sanders J, et al. WRISK voices: A mixed-methods study of women's experiences of pregnancy-related public health advice and risk messages in the UK. *Midwifery* 2022;113:103433. doi: https://doi.org/10.1016/j.midw.2022.103433
16. Parliamentary office of Science and technology. Infant Mortality and Stillbirth in the UK POSTnote 2016.
17. The NHS Race & Health Observatory. The Power of Language: a consultation report on the use of collective terminology at the NHS Race & Health Observatory 2021 [Available from:

For peer review only

https://www.nhsrho.org/wp-content/uploads/2021/11/NHS_RaceHealthObservatory_Terminology-consultation-report-NOV-21-1.pdf accessed 18 August 2022.
18. Braun V, Clarke V. Using thematic analysis in psychology. *Qualitative Research in Psychology* 2006;3(2):77-101. doi: 10.1191/1478088706qp063oa
19. Salmona M, Lieber E, Kaczynski D. Qualitative and mixed methods data analysis using Dedoose: A practical approach for research across the social sciences: Sage Publications 2019.
20. Microsoft Corporation. Microsoft Excel 2018 [Available from: https://office.microsoft.com/excel accessed 24 August 2022.
21. StataCorp. Stata Statistical Software: Release 15. College Station, TX: StataCorp LLC; 2017 [
22. National Institute for Health and Care Excellence. Diabetes in pregnancy: management from preconception to the postnatal period 2020 [
23. Gynaecologists RCoOa. Epilepsy In pregnancy Greentop Guideline 68. London, 2016.
24. Shroukh WA, Steinke DT, Willis SC. Risk management of teratogenic medicines: A systematic review. *Birth Defects Res* 2020;112(20):1755-86. doi: 10.1002/bdr2.1799 [published Online First: 2020/09/13]
25. England PH. BUMPS The best use of medicines in pregnancy 2022 [Available from: https://www.medicinesinpregnancy.org/Medicine--pregnancy/ accessed 24 August 2022.
26. Wise J. Pregnant women should be included in clinical trials to improve outcomes, says commission. *Bmj* 2022;377:o1193. doi: 10.1136/bmj.o1193
27. McBride WG. Thalidomide and congenital abnormalities. *The Lancet* 1961;278(7216):1358. doi: https://doi.org/10.1016/S0140-6736(61)90927-8
28. Petersen I, McCrea RL, Lupattelli A, et al. Women's perception of risks of adverse fetal pregnancy outcomes: a large-scale multinational survey. *BMJ Open* 2015;5(6):e007390. doi: 10.1136/bmjopen-2014-007390
29. Vousden N, Ramakrishnan R, Bunch K, et al. Management and implications of severe COVID-19 in pregnancy in the UK: data from the UK Obstetric Surveillance System national cohort. *Acta Obstetricia et Gynecologica Scandinavica* 2022;101(4):461-70. doi: https://doi.org/10.1111/aogs.14329

For peer review only

For peer review only

COREQ (COnsolidated criteria for REporting Qualitative research) Checklist

A checklist of items that should be included in reports of qualitative research. You must report the page number in your manuscript where you consider each of the items listed in this checklist. If you have not included this information, either revise your manuscript accordingly before submitting or note N/A.

Topic	Item No.	Guide Questions/Description	Reported on Page No.
**Domain 1: Research team and reflexivity**			
Personal characteristics			
Interviewer/facilitator	1	Which author/s conducted the interview or focus group?	
Credentials	2	What were the researcher's credentials? E.g. PhD, MD	
Occupation	3	What was their occupation at the time of the study?	
Gender	4	Was the researcher male or female?	
Experience and training	5	What experience or training did the researcher have?	
Relationship with participants			
Relationship established	6	Was a relationship established prior to study commencement?	
Participant knowledge of the interviewer	7	What did the participants know about the researcher? e.g. personal goals, reasons for doing the research	
Interviewer characteristics	8	What characteristics were reported about the inter viewer/facilitator? e.g. Bias, assumptions, reasons and interests in the research topic	
**Domain 2: Study design**			
Theoretical framework			
Methodological orientation and Theory	9	What methodological orientation was stated to underpin the study? e.g. grounded theory, discourse analysis, ethnography, phenomenology, content analysis	
Participant selection			
Sampling	10	How were participants selected? e.g. purposive, convenience, consecutive, snowball	
Method of approach	11	How were participants approached? e.g. face-to-face, telephone, mail, email	
Sample size	12	How many participants were in the study?	
Non-participation	13	How many people refused to participate or dropped out? Reasons?	
Setting			
Setting of data collection	14	Where was the data collected? e.g. home, clinic, workplace	
Presence of non-participants	15	Was anyone else present besides the participants and researchers?	
Description of sample	16	What are the important characteristics of the sample? e.g. demographic data, date	
Data collection			
Interview guide	17	Were questions, prompts, guides provided by the authors? Was it pilot tested?	
Repeat interviews	18	Were repeat inter views carried out? If yes, how many?	
Audio/visual recording	19	Did the research use audio or visual recording to collect the data?	
Field notes	20	Were field notes made during and/or after the inter view or focus group?	
Duration	21	What was the duration of the inter views or focus group?	
Data saturation	22	Was data saturation discussed?	
Transcripts returned	23	Were transcripts returned to participants for comment and/or	

For peer review only

For peer review only - http://bmjopen.bmj.com/site/about/guidelines.xhtml

Topic	Item No.	Guide Questions/Description	Reported on Page No.
		correction?	
**Domain 3: analysis and findings**			
Data analysis			
Number of data coders	24	How many data coders coded the data?	
Description of the coding tree	25	Did authors provide a description of the coding tree?	
Derivation of themes	26	Were themes identified in advance or derived from the data?	
Software	27	What software, if applicable, was used to manage the data?	
Participant checking	28	Did participants provide feedback on the findings?	
Reporting			
Quotations presented	29	Were participant quotations presented to illustrate the themes/findings? Was each quotation identified? e.g. participant number	
Data and findings consistent	30	Was there consistency between the data presented and the findings?	
Clarity of major themes	31	Were major themes clearly presented in the findings?	
Clarity of minor themes	32	Is there a description of diverse cases or discussion of minor themes?	

Developed from: Tong A, Sainsbury P, Craig J. Consolidated criteria for reporting qualitative research (COREQ): a 32-item checklist for interviews and focus groups. *International Journal for Quality in Health Care*. 2007. Volume 19, Number 6: pp. 349 – 357

Once you have completed this checklist, please save a copy and upload it as part of your submission. DO NOT include this checklist as part of the main manuscript document. It must be uploaded as a separate file.

For peer review only

BMJ Open

Women's experiences of over the counter and prescription medication during pregnancy in the UK: findings from survey free-text responses and narrative interviews.

Journal:	*BMJ Open*
Manuscript ID	bmjopen-2022-067987.R1
Article Type:	Original research
Date Submitted by the Author:	30-Nov-2022
Complete List of Authors:	Sanders, Julia; Cardiff University, School of Healthcare Sciences<br>Blaylock, Rebecca; British Pregnancy Advisory Service, Centre for Reproductive Research & Communication<br>Dean, Caitlin; Pregnancy Sickness Support<br>Petersen, Irene; University College London Medical School, Department of Primary Care and Population health<br>Trickey, Heather; University of Cardiff, Social Medicine<br>Murphy, Clare; British Pregnancy Advisory Service, Centre for Reproductive Research & Communication
**Primary Subject Heading**:	Obstetrics and gynaecology
Secondary Subject Heading:	General practice / Family practice, Obstetrics and gynaecology, Pharmacology and therapeutics, Qualitative research, Reproductive medicine
Keywords:	CLINICAL PHARMACOLOGY, Reproductive medicine < GYNAECOLOGY, Maternal medicine < OBSTETRICS, PUBLIC HEALTH, QUALITATIVE RESEARCH

SCHOLARONE™
Manuscripts

I, the Submitting Author has the right to grant and does grant on behalf of all authors of the Work (as defined in the below author licence), an exclusive licence and/or a non-exclusive licence for contributions from authors who are: i) UK Crown employees; ii) where BMJ has agreed a CC-BY licence shall apply, and/or iii) in accordance with the terms applicable for US Federal Government officers or employees acting as part of their official duties; on a worldwide, perpetual, irrevocable, royalty-free basis to BMJ Publishing Group Ltd ("BMJ") its licensees and where the relevant Journal is co-owned by BMJ to the co-owners of the Journal, to publish the Work in this journal and any other BMJ products and to exploit all rights, as set out in our licence.

The Submitting Author accepts and understands that any supply made under these terms is made by BMJ to the Submitting Author unless you are acting as an employee on behalf of your employer or a postgraduate student of an affiliated institution which is paying any applicable article publishing charge ("APC") for Open Access articles. Where the Submitting Author wishes to make the Work available on an Open Access basis (and intends to pay the relevant APC), the terms of reuse of such Open Access shall be governed by a Creative Commons licence – details of these licences and which Creative Commons licence will apply to this Work are set out in our licence referred to above.

Other than as permitted in any relevant BMJ Author's Self Archiving Policies, I confirm this Work has not been accepted for publication elsewhere, is not being considered for publication elsewhere and does not duplicate material already published. I confirm all authors consent to publication of this Work and authorise the granting of this licence.

For peer review only

Title

Women's experiences of over the counter and prescription medication during pregnancy in the UK: findings from survey free-text responses and narrative interviews.

Julia Sanders[a]
Rebecca Blaylock[b]
Caitlin Dean[c]
Irene Petersen[d]
Heather Trickey[e]
Clare Murphy[b]

[a] School of Healthcare Sciences, Cardiff University, United Kingdom
[b] Centre for Reproductive Research & Communication, British Pregnancy Advisory Service, 30-31 Furnival Street, London, United Kingdom
[c] Pregnancy Sickness Support, 19G Normandy Way, Bodmin, United Kingdom
[d] Department of Primary Care & Population Health, University College London, United Kingdom
[e] School of Social Sciences, Cardiff University, United Kingdom

Emails and ORCID IDs

Julia Sanders sandersj3@cardiff.ac.uk 0000-0001-5712-9989
Rebecca Blaylock rebecca.blaylock@bpas.org 0000-0003-4317-1638
Caitlin Dean caitlin@pregnancysicknesssupport.org.uk 0000-0002-8812-5101
Irene Petersen i.petersen@ucl.ac.uk 0000-0003-2446-0506
Clare Murphy clare.murphy@bpas.org 0000-0002-7272-9605

Corresponding author

Rebecca Blaylock
Research & Engagement Lead
Centre for Reproductive Research & Communication
British Pregnancy Advisory Service
30-31 Furnival Street
London
EC4A 1JQ
Email: rebecca.blaylock@bpas.org

For peer review only

References: 30

Abstract word count: 207

Word count: 4341

Abstract

Objectives: To explore women's experiences of over the counter and prescription medication advice and use during pregnancy.

Design: A study design consisting of an online survey and nested in-depth interviews with a sub-sample of participants. We analysed data from survey free-text responses and in-depth interviews using thematic analysis. Quantitative survey data is published elsewhere.

Setting: The United Kingdom.

Participants: A total of 7090 women completed the survey, and 34 women who collectively had experienced 68 pregnancies were subsequently interviewed. Women were eligible if living in the UK, aged 16-45 years, were pregnant or had been pregnant in the last five years regardless of pregnancy outcome.

Results: Medication prescribing and use during pregnancy was common. The prescribing, dispensing, and taking of some advised medications was restricted through women's or prescribers' fear of fetal harm. Lack of adherence to national prescribing guidance, conflicting professional opinion, and poor communication resulted in maternal anxiety, avoidable morbidity, and women negotiating complex and distressing pathways to obtain recommended medications. In contrast, some women felt overmedicated and that pharmacological treatments were used without exploring other options first.

Conclusions: Increased translation of national guidance into practice, and greater personalisation of antenatal care are needed to improve the safety, efficacy, and personalisation of prescribing in pregnancy.

Strengths and limitations of this study

- The study gained new insights into a seldom researched topic
- A sampling frame ensured inclusion of women with social and medical complexities from across the UK
- Women without sufficient spoken English or internet access were excluded
- Interviews were conducted only with women, not the health professionals who prescribe

Title

Women's experiences of over the counter and prescription medication during pregnancy in the UK: findings from survey free-text responses and narrative interviews.

Background

Safe and effective prescribing is an essential component of antenatal care. Prescribing in pregnancy requires additional knowledge and caution due to the potential for teratogenesis, altered pharmacokinetics, maternal concerns[1], and potential for short and longer term harm to the fetus[2, 3]. Antenatal information[4] provided to pregnant women states that most medications reach the fetus and all medication use should be discussed with health professionals. Many women discontinue or avoid medications in pregnancy[5], however, lack of treatment can potentially have severe consequences[1]. Despite selective avoidance, medication use in pregnancy is common with around 87% of pregnant women in the UK reporting medication use for short term or chronic conditions[6]. Excluding vitamin and iron supplements, frequently prescribed and over the counter (OTC) medications taken during pregnancy include antacids, analgesics, anti-emetics, and antibiotics[6].

There are few medications that should not be used by pregnant women, for example thalidomide, sodium valproate, and isotretinoin due to their teratogenicity. Most other medications are safe and widely used during pregnancy and there are several National prescribing guidelines[7, 8] for treatment during pregnancy. For example, the National

peer review only

Institute for Health and Care Excellence (NICE) guidance on antenatal and postnatal mental health recommend that health professionals should discuss the potential benefits of psychological interventions and psychotropic medication, the possible consequences of no treatment and possible harms associated with treatment and what might happen if treatment is changed or stopped, particularly if psychotropic medication is stopped abruptly[8]. For mild to moderate mental health problems psychological interventions should form first line management[8], but access is limited by long waiting lists and high referral thresholds, whilst a lack of continuity in antenatal care leads to difficulty developing trusting therapeutic relationships[9]. Suicide is the leading cause of direct maternal death in the first postnatal year[10] , and reports have highlighted that treatments for depression may often be discontinued in pregnancy[5, 11].

The Royal College of Obstetricians and Gynaecologists have set out clear guidance on the diagnosis and subsequent management of nausea and vomiting in pregnancy across community, ambulatory day-care, and inpatient settings, including a medication escalation ladder for use when first line recommended treatments are ineffective[7].

There is growing concern that public health messages aimed at pregnant women, including those relating to medication use, do not always fully reflect or explain the evidence base underpinning them and the nuances and complexity of information is lost[12]. Thhe inability to receive effective medications is not without consequence for women and babies. A survey in 2015 identified women with severe hyperemesis gravidarum frequently had difficulty obtaining swift treatment and support for debilitating pregnancy sickness resulting in some terminating an otherwise wanted pregnancy[13]. Likewise, although mental health problems are common during pregnancy[14] previous reports have highlighted barriers to access to appropriate treatment[15].

The 'WRISK project: Understanding and improving the way risk in pregnancy is communicated to women' was established to explore women's experiences of maternity related public health and risk messaging, including those relating to medication use in pregnancy. The study aimed to hear women's voices through public involvement, quantitative, and qualitative methodologies with a specific objective of exploring the views of women previously identified as feeling stigmatised or poorly served by current

practice[16] . This paper focuses specifically on women's experiences of medication use in pregnancy as reported in open text comments in the survey and in-depth interviews.

Methods

Study design

The study design consisted of an online survey and nested in-depth interviews with a sub-sample of survey participants. The survey was open to UK residents aged 16-45 years, who were pregnant or who had been pregnant in the last five years, regardless of pregnancy outcome. The survey was publicised through social media platforms including those of the study's charity partners. We invited survey respondents to express interest in further research involvement and recruited participants for the interviews from this population. As the survey was exploratory, an a priori sample size calculation was not required.

For subsequent in-depth interviews we used a sampling frame to ensure we heard the voices of women at greater risk of the most severe pregnancy outcomes, maternal death or stillbirth[11, 17]. The sampling frame aimed to ensure that 30-35 women were interviewed and included a minimum of 20% who were eligible for means-tested state benefits, a minimum of 20% from Black, Asian, and Minority Ethnic backgrounds, and at least six women were interviewed with experience of the following pregnancy experiences: pregnancy <20 years of age, Body Mass Index >30Kg/m^2, antenatal mental health problems or experience of hyperemesis gravidarum. Interested respondents who met the sampling criteria were selected using a random number table, until required numbers were obtained.

Data Collection

Survey

A participant information sheet and consent form were integrated into the survey. The survey included questions on participants' experience of the advice, information, and support they received from different sources during pregnancy, with the focus on the respondent's most recent pregnancy. Questions with open free-text responses were included alongside questions with Likert scales and multiple-choice responses. We describe the survey and demographic characteristics of respondents in full in a previous

For peer review only

publication[16]. For the purposes of this publication, we present free text responses from the survey pertaining to medication use in pregnancy only.

One-to-one interviews

The participant information sheets, consent forms, and topic guides for the interviews were developed in collaboration with the combined researcher and charity project advisory group. Narrative topic guides allowed participants to tell their pregnancy stories and were tailored to various pregnancy outcomes. Participants were asked to primarily discuss their most recent pregnancy and were asked about antenatal prescribing and use of over-the-counter medications, amongst other topics.

Participants were offered the option of in-person or telephone interviews. Interviewers [RB and HT] made detailed field notes following each interview. Participants were offered, and all received a high street vouchers worth £20. Audio files were transcribed verbatim using a commercial transcription service. Electronic transcripts were stored separately to any identifiable data on a secure IT system, and audio files deleted once they had been checked for accuracy.

Research team

The trained, all female research team had extensive experience of working in pregnancy-related research and practice including social sciences (RB and HT), medical law (JS), public health (RB, JS, and HT) and clinical midwifery (JS). Interviews were conducted and analysed by RB and HT.

Terminology

The WRISK project was inclusive of all people who had experienced pregnancy in the previous five years. The project team always referred to individuals according to their self-determined gender. In this paper we use the words ‘woman/ women’ as the vast majority of participants self-identified as women. We use the term “BAME”, but acknowledge that this is problematic[18].

For peer review only

Data analysis

The interviews and free text comments in the survey were analysed thematically following Braun and Clarke's method[19]. Transcripts were coded and analysed using Dedoose[20] by RB and HT. All transcripts and accompanying data such as researcher's field notes were read in detail several times by both interviewers, and high-level codes pertaining to the original research questions were identified. Further codes were identified in the data during an inductive process, resulting in a coding framework informed by the data itself. Narrative summaries of data pertaining to each code were produced and cross-checked by RB and HT, and subsequently organised into high-level themes. We used Excel® and STATA® to generate descriptive statistics on the survey and interview populations[21] [22].

Patient and Public Involvement

The project oversight group included representatives from five diverse maternity user groups, healthcare professionals, and researchers in addition to the study team. The group met regularly throughout the project from conception to dissemination and collectively informed all aspects of study design and delivery.

Results

The survey was completed by 7090 women, of whom 3,175 (44.1%) expressed willingness for further involvement in research, and 34 were subsequently interviewed. Interviews were carried out by RB and HT between July and November of 2019, lasting approximately 45-60 minutes. Two pilot interviews were conducted in-person in April 2019 and were deemed to be of sufficient quality to be included in the analysis. All subsequent interviews were conducted by telephone and recorded using a Dictaphone. The 34 women interviewed included those with experience of: a BMI >30, (n=7); antenatal medication use for mental health conditions, (n=6); medication for hyperemesis gravidarum, (n=9); age <20 during pregnancy, (n=7); or having a termination due to a perceived or actual risk either to themselves or their baby (n=7). Some participants fitted more than one category, whilst five had none of these experiences.

For peer review only - http://bmjopen.bmj.com/site/about/guidelines.xhtml

Sociodemographic characteristics of those interviewed are described in Table 1. Sociodemographic characteristics of all survey respondents and quantitative survey findings are published elsewhere[16].

Table 1

Sociodemographic characteristics of interview participants at time of interview

SOCIODEMOGRAPHICS	N (34)	%
Age		
**16-18**	0	0
**19-20**	2	5.9
**21-25**	9	26.5
**26-30**	7	20.6
**31-35**	7	20.6
**36-40**	7	20.6
**41-45**	0	0
**45+**	1	2.9
**Missing**	1	2.9
Highest level of education		
**Secondary school**	2	5.9
**Apprenticeship/HND/NVQ**	3	8.8
**A-Levels**	6	17.6
**Undergraduate degree**	13	38.2
**Postgraduate degree**	10	29.4
**Missing**	0	0
Relationship status		
**Married**	23	67.6
**Have a partner & live with them**	9	26.5
**Have a partner & live separately**	1	2.9
**Polyamorous**	1	2.9
Ethnicity		
**White: English/Welsh/Scottish/Northern Irish/British**	24	70.6
**Black/African/Caribbean/ Black British: African**	3	8.8

For peer review only

**Mixed/multiple ethnic groups: White & Black African**	1	2.9
**Asian/Asian British: Chinese**	1	2.9
**Asian/Asian British: Indian**	1	2.9
**Black/African/Caribbean/ Black British: Caribbean**	1	2.9
**Mixed/multiple ethnic groups: White & Black Caribbean**	2	5.9
**Mixed/multiple ethnic groups: White British & Middle Eastern**	1	2.9
Gender		
Female	33	97.1
Non-binary	1	2.9
Receive State benefits?		
**Yes**	10	29.4
**No**	23	67.6
**Missing**	1	2.9
**PREGNANCY HISTORY**	**Mean**	**Range**
Gravidity	2	1-5
Live births	1.2	1-4
Terminations/abortion	0.3	0-2
Miscarriage/stillbirth	0.3	0-2

Four themes were identified in relation to medication use in pregnancy: ‘fear of medications and self-regulation’, ‘feeling overmedicated’, ‘conflicting opinions’, and ‘running the gauntlet’. Some themes related to women’s experiences of medication use in general, while others were condition specific.

Fear of medications and self-regulation

Fear, or anxiety for the potential for fetal harm caused by over the counter and prescribed medications were expressed by women. They also reported prescribers’ reluctance to prescribe for this reason. Participants expressed support for the precautionary approach that medications should be kept to a minimum during pregnancy. The fear of causing fetal harm through taking medication resulted in women reducing medication even when this was required to control serious medical conditions.

Two women described the consequences of stopping prescribed medication for pre-existing medical conditions. One woman stopped taking her asthma medication which resulted in hospitalisation:

For peer review only

"I stopped taking my inhaler, just because I just didn't want to harm the baby. I took a bad cold, and I was coughing up blood, and then they sent me to hospital for all these other tests. They thought I had a clot or something. So that obviously scared me. Then I just said, Screw it. I'm just going to take my asthma medication, and that was that." [WRI28]

Similarly, another woman experienced a serious migraine and temporary blindness, following discontinuation of medication for cranial hypertension:

"I have a brain condition called... Well, it's cranial hypertension, so it just means that my body produces too much spinal fluid, which pushes on my brain, which causes migraines, essentially. … because there isn't a lot of research into this condition in pregnancy, I decided, personally, to stop taking the medication. Yes, so I stopped all my medication and, thankfully, touch wood, everything was fine. … I did struggle with a few migraines throughout the pregnancy, one which was quite severe, which resulted in a loss of vision in my left eye, but luckily, it came back. So that was fine. That was quite difficult to deal with, though." [WRI23]

Self-regulation of medication particularly related to the use of analgesics. Women described conflict between their understanding of some medications being safe to take in pregnancy, with their desire to try "not to take them" [WRI11] due to the possibility of potential risks to the fetus.

"… we all know it's [paracetamol] is generally safe, it's one of the safest painkillers there is, so it's the only one you can take, but even just taking that, women don't like- you know, they have a headache instead of taking the medicine, because they feel so guilty." [WRI30]

Another woman described managing with paracetamol for pelvic girdle pain even after she had been prescribed codeine-based medication:

"Yes, one of those [codeine-based analgesia] and I was reading the side effects and I was like, 'No, I'm not happy taking it and having that go into

For peer review only

my baby.' I just literally was taking paracetamol and I was just trying to take the lowest dose of paracetamol that I could really." [WRI4]

Whilst women generally tried to avoid prescribed medication, recommended vaccines, including for influenza, appeared to be accepted. Supplements were viewed as positive and often taken without any apparent fear of causing an adverse pregnancy outcome.

"Interviewer: Did you take medications while you were pregnant?

No, not at all.

Interviewer: No, not at all. Okay.

Beyond supplements and the flu jab and recommended stuff like that. I had a whooping cough jab, I think, with [daughter's name] but no regular medication of any sort." (WRI7)

There were many examples of health professionals reinforcing the message that medication should be avoided in pregnancy.

"Throughout my pregnancy I really struggled with my mental health. So, at one point, I don't know how pregnant I was, I must have been about six or seven months, we got an emergency appointment at my GP's surgery… and we said, 'I'm really struggling. Is there anything you can do?' His first reaction was that I shouldn't be on any medication because I was pregnant." [WRI29]

Health professionals expressing their own concerns around prescribing had a particularly negative impact on women. One interviewee [WRI17] explained her concern when a hospital consultant prescribing "put his hands together in prayer and said, '*God, forgive me for giving you this. I hope your baby's okay'*" when prescribing ondansetron for severe hyperemesis gravidarum (HG).

Other women had similar experiences when accessing medication for their mental health. WRI33 said:

"because I was being so sick, I went to the doctor's about anti-sickness medication. She saw I was on antidepressants and she made me feel like I was the world's worst

For peer review only

mother. I don't know whether she meant to, but she was just worried that, 'Ooh, you're pregnant and you're on medication.' She made me feel like, 'You're hurting your baby'. Then when I went to the pharmacy to pick up my meds, they made me feel like it on the same day as well. I think that was the point where I started to really decline in my mental health. It just seemed to trigger something off that I was going to be a terrible mother."

Feeling overmedicated

Some participants reported feeling overmedicated, and that pharmacological treatments were used without exploring other options first. This was the case for women with both mental and physical health concerns:

"I feel like they push drugs on people too quickly, that is just the go-to. I had a traumatic birth with number two. That's why I went on the antidepressants with number three, because I was terrified of giving birth. They just kept telling me baby was getting so big, blah blah blah. I was, literally, terrified of giving birth to her. They just put my antidepressants. There was no, 'Let's talk about it, get counselling, and get over the traumatic birth.' It was just mask it really. I don't think that's right to do when you're dealing with, obviously, a pregnant woman, a baby. [WRI31]

Current guidance relating to gestational diabetes recommends metformin is introduced if good blood glucose control is not achieved with 1 – 2 weeks of dietary change and exercise[23]. One survey respondent would have preferred to extend this duration before commencing medication.

"I would have liked to have been better involved in decision making around my options for treatment in relation to my gestational diabetes… I ended up with 2 tablets of metformin which I think was overkill - I would have liked to have had the opportunity to try harder with diet and exercise before starting medication."

For peer review only

Conflicting opinions

Women who required prescribed medication in pregnancy described frustration and distress resulting from conflicting opinions of health professionals. This conflict was particularly felt by women with hyperemesis gravidarum or mental health problems. One woman who required ondansetron for severe hyperemesis gravidarum was prescribed it, later to have this decision questioned by another General Practitioner [GP] in the same practice:

> *"So, I was prescribed by one doctor the ondansetron and because I was running out, they said I had to speak to another doctor just to check it was obviously working, just a check-up on it, to get the repeat prescription. Then when this doctor phoned me, she said, 'You shouldn't be taking this. This is for cancer patients. There's no reason why you should be taking it. You're putting your baby's life at risk'. … She just wasn't having any of it. I even told her that I'd just had a scan like 45 minutes before the phone call, and she said, 'Oh well, that can change at your 20 week scan. Don't come crying to me when they tell you that your baby has got heart defects'".*
>
> *[WRI15]*

Another participant described the distress that resulted when a pharmacist would not fulfil a prescription for anti-depressants prescribed by her GP.

> *"They just wouldn't give me my prescription, and people were behind me in the queue and could hear what I was on, that I was pregnant. I wasn't 12 weeks yet, so I hadn't told anybody. I had to just sit there waiting for them to decide whether I could have it or not and then they said, 'No, you can't have it.' I left without any medication, and I was just crying in the car."*
>
> *[WRI33]*

Two interview participants said that conflicting and contradictory advice from healthcare professionals was "staggering".

For peer review only

"I found through everything in my pregnancy that all the doctors I saw had completely different advice and completely different opinions to each other..." [WRI6]

The conflict was seen across all professional groups. One respondent described conflicting information from her midwife and GP.

"I was advised by my GP to continue my anti-depressant medication. My midwife continually questioned this decision throughout the whole pregnancy". [Survey respondent]

Running the Gauntlet

This theme particularly related to prescribing for hyperemesis gravidarum. Some women experienced prolonged periods prior to getting effective treatment. Four women who were suffering with hyperemesis gravidarum described their experience of the current guidance.

*"they tried me on cyclizine, which just made me dizzy and did nothing for the sickness. I went very dizzy and sleepy. And then Stemetil… Which also had no effect. So, I was, kind of, getting desperate. And then, … they gave me ondansetron which is what they give to chemo patients, you probably know, and it was like I was alive again. … They were sympathetic, but I didn't get a lot of advice on what effect it would have on the baby. Just, you know, like, with the Cyclzine and the Stemetil, they were very much, like, 'It's been used a lot and we can be certain from observations that it won't have any effect.' So that was fine. They were, kind of, reluctant to, like, move me onto the stronger stuff." [**WRI1]***

"… they could see that I couldn't even take a sip of water without being sick, so I needed something. So, first off, I was given a tablet that you put between your gum and your teeth. It wasn't working. So, I went back and I got something called cyclizine, which is a tablet. That started to make it a little bit better for a couple of weeks and then I just got worse again. So, I had to go back again and …[they] gave me a pill called ondansetron.

For peer review only

Then that was working, but I was still quite bad. So, I went back again and they gave me a combination of cyclizine and ondansetron, and that did the trick in the end". WRI6

Even when women were prescribed treatment for hyperemesis, they could then be discouraged from taking it by the prescriber:

"The most unhelpful piece of advice I received was from a doctor, who prescribed me anti-sickness tablets for hyperemesis but said 'try not to take them unless you feel you need them' because 'we can't say that any medicine is safe in pregnancy'. I was very unwell, very dehydrated and very disoriented and was afraid of causing harm to the baby so I didn't take them." [Survey respondent]

Another woman who was prescribed medication received comments from a pharmacist who suggested they may be unnecessary:

"My husband went to collect my prescription when I was discharged, and was told that I was being 'irresponsible' and 'putting the baby at risk' by taking the medication. Later in pregnancy (I had HG until the moment of delivery), another pharmacist told me that I shouldn't still be taking medication as morning sickness would've stopped by my late stage, implied it was all in my head, and suggested that I try a herbal remedy instead." [Survey respondent]

For peer review only

Discussion

This study adds important understanding to the seldom explored topic of women's experiences of antenatal medication use. Many women wished to reduce exposure to over the counter and prescribed medication use during pregnancy, but dietary supplements and vaccines, were generally accepted by pregnant women. This contrasted to treatments for mental health problems and hyperemesis gravidarum, conditions where pregnant women described having to negotiate conflicting opinions of health professionals to obtain recommended or effective treatments. Too often prescribing was more restrictive than recommended in national guidance resulting in avoidable, or prolonged, maternal morbidity, distress, and anxiety. Similar to the lack of preconceptual and antenatal care for women with epilepsy highlighted in reviews of maternal deaths[11], where information on medications used for chronic conditions was not shared, some women discontinued treatments without medical consultation, resulting in hospitalisation or exacerbation of symptoms.

In the UK, prescribing for pregnant women is undertaken by different health professionals which complicates communication. Women with existing medical conditions, including epilepsy, require preconceptual advice on medication use[24]. GPs are frequently involved in prescribing decisions, commonly prescribed medications such as iron therapy and low dose aspirin may be prescribed and administered by midwives. Women with additional obstetric needs will receive care led by obstetricians, who may share prescribing with GPs or specialist physicians. This arena is further complicated by public health bodies who are responsible for producing public health risk messages for pregnancy being independent from both primary and secondary care where prescribing occurs. This multidisciplinary approach to antenatal prescribing was found often to be fragmented, with women hearing conflicting opinions even from different members of the same professional group. The role of pharmacists as medication 'gatekeepers' and their refusal to dispense prescribed medications highlights the importance of their inclusion in system improvements.

Even when effective treatments were endorsed in national guidance, challenges in implementation were found. Despite RCOG guidance on the management of hyperemesis gravidarum[7], women reported being denied access to effective treatments. Ensuring

For peer review only

health professionals have easy access to up-to-date guidance on specialist aspects of care, and sufficient time and support to incorporate guidance into their practice is imperative. Whilst prescribers need to balance maternal benefit with potential fetal harm when prescribing in pregnancy, women's individual circumstances were not always considered, and they were not fully engaged in decision making. Possibly reflecting the tendency of health professionals to overestimate the teratogenic potential of drugs[25] we found many examples where health professionals used fear of fetal harm to justify a refusal to prescribe or dispense otherwise recommended medications. This had a significant impact not only on women's health, but on their emotional wellbeing with one reporting they were made to feel like the "world's worst mother".

Some women felt antidepressants and metformin for gestational diabetes were offered in preference to non-pharmacological options. This may reflect that the availability of talking therapies does not meet demand, or in relation to gestational diabetes, a lack of informed personalised conversations on the effectiveness of methods to obtain glycaemic control.

High quality, easily accessible information on the safety of medicines is available[26] but appeared to be underutilised in informing individualised discussions. Better reporting systems on outcomes related to medication use in pregnancy are needed, including making the results of clinical trials more generalisable through, where appropriate, the inclusion of pregnant women[27]. Minimising prescribing in pregnancy became deeply entrenched in the ethos of antenatal care following the thalidomide tragedy[28]. The belief that all medication use during pregnancy carries risk is commonly held among women, with paracetamol, antibiotics and antidepressants[29] considered to be on a continuum of increasing risk. The potential for this position to cause harm is increasingly being realised. Some women with epilepsy, and others with serious mental health conditions have died because of an over stringent position on medication avoidance[11]. More recently the numbers of pregnant women who died during the covid-19 pandemic could have been reduced through earlier acceptance of the safety of vaccination in pregnancy, better public health communication, and greater use of effective treatments among those seriously ill with COVID-19[30]. While the need to reduce maternal mortalities through improved prescribing is already appreciated, our study suggests physical and mental morbidity caused

For peer review only

through the lack of access to effective treatments for mental health conditions and hyperemesis gravidarum is likely to be very common and requires improvement.

Strengths of this study include adding to knowledge on a seldom explored topic, the geographical spread and high number of survey participants from across the UK, and use of a sampling frame for the selection of interview participants. A weakness was that the sampling frame design may have shaped our findings and focused attention on certain experiences of medication use at the expense of others of equal concern to women. Our survey was self-selecting and may reflect the views of those more motivated to participate in research. The survey was only available in English and via the internet, excluding some groups from participation including non-English reading women, and those with limited or no access to the internet. This study would have been strengthened by the inclusion of clinicians to better understand their attitudes and understandings of prescribing and dispensing during pregnancy.

Conclusion

Medication prescribing and use during pregnancy is common. However, fear of fetal harm restricts the prescribing and taking of some advised medications. Analgesics were commonly avoided or taken at lower than the therapeutic or prescribed dose and some women reduced or discontinued medications for chronic conditions without medical oversight. Where existing clinical guidance was not followed, or there was conflict in professional opinion, reluctance to prescribe or dispense resulted in women needing to negotiate complex and distressing pathways to obtain required medications. The study identified aspects of antenatal prescribing where improvement in knowledge, communication, or practice is required to ensure maximisation of the safety, efficacy, and personalisation of prescribing in pregnancy.

For peer review only

Funding statement

This work was supported by the Wellcome Trust as part of the WRISK Project [212089/Z/18/Z].

Data availability statement

Data are available on request.

Ethics statements

Patient consent for publication

Not applicable.

Ethical approval

Ethical approval was granted by the Research and Ethics committee of the School of Social Sciences at Cardiff University SREC/3201.

Competing interests

None

Author Contributions

CM and HT conceptualised the study and secured funding. CM, RB, HT, JS, IP and CD developed the protocol. HT and RB conducted the data collection and analysis. CM, JS, HT and RB provided study management. JS drafted the original manuscript. RB, IP and CD contributed to writing. CD and IP were members of the project advisory group. All authors approved the final manuscript.

Acknowledgements

We acknowledge and thank the group of over 7,000 women who shared their intimate stories of pregnancy with us. Special thanks go to the Oversight Committee: Dame Cathy Warwick, BPAS Chair of Board of Trustees and Chair of the Oversight Committee; Rebecca Brione, Birthrights; Jane Fisher, Antenatal Results and Choices; Caitlin Dean, Pregnancy Sickness Support; Elizabeth Duff, NCT; Professor Irene Petersen, University College London; Amber Marshall, BigBirthas.co.uk; Professor Fiona Woollard, Southampton University; Rolling representative, Public Health Wales.

We dedicate this paper to the late Dr Heather Trickey, without whom the project would never exist. Thank you for your inspiration, and dedication to the health and wellbeing of all women.

For peer review only

For peer review only

References

[1] M. J. Twigg, A. Lupattelli, and H. Nordeng, "Women's beliefs about medication use during their pregnancy: a UK perspective," (in eng), *Int J Clin Pharm,* vol. 38, no. 4, pp. 968-976, 2016, doi: 10.1007/s11096-016-0322-5.

[2] W. H. Meeraus, I. Petersen, and R. Gilbert, "Association between antibiotic prescribing in pregnancy and cerebral palsy or epilepsy in children born at term: a cohort study using the health improvement network," *PLoS ONE [Electronic Resource],* Research Support, Non-U.S. Gov't vol. 10, no. 3, p. e0122034, 2015. [Online].

[3] L. Page, "Prescribing for pregnancy: managing prescribing for women with mental health diagnoses," *Drug & Therapeutics Bulletin,* Review vol. 58, no. 1, pp. 8-11, 2020. [Online].

[4] N. England. "Medicines in pregnancy." https://www.nhs.uk/pregnancy/keeping-well/medicines/ (accessed 24 August 2022.

[5] I. Petersen, R. E. Gilbert, S. J. Evans, S. L. Man, and I. Nazareth, "Pregnancy as a major determinant for discontinuation of antidepressants: an analysis of data from The Health Improvement Network," (in eng), *J Clin Psychiatry,* vol. 72, no. 7, pp. 979-85, Jul 2011, doi: 10.4088/JCP.10m06090blu.

[6] A. Lupattelli *et al.*, "Medication use in pregnancy: a cross-sectional, multinational web-based study," *BMJ Open,* vol. 4, no. 2, p. e004365, 2014, doi: 10.1136/bmjopen-2013-004365.

[7] Royal College of Obstetricians and Gynaecologists, "The Management of Nausea and Vomiting of Pregnancy and Hyperemesis Gravidarum," in "Green-top Guideline No. 69," London, 2016.

[8] National Insitute for Health and Care Excellence, "Antenatal and postnatal mental health: clinical management and service guidance. Clinical Guideline 192," 2020. [Online]. Available: www.nice.org.uk/guidance/cg192

[9] O. Megnin-Viggars, I. Symington, L. M. Howard, and S. Pilling, "Experience of care for mental health problems in the antenatal or postnatal period for women in the UK: a systematic review and meta-synthesis of qualitative research," (in eng), *Arch Womens Ment Health,* vol. 18, no. 6, pp. 745-59, Dec 2015, doi: 10.1007/s00737-015-0548-6.

[10] B. K. Knight M, Tuffnell D, Patel R, Shakespeare J, Kotnis R, Kenyon S, Kurinczuk JJ (Eds.) on behalf of MBRRACE-UK,, "Saving Lives, Improving Mothers' Care - Lessons learned to inform maternity care from the UK and Ireland Confidential Enquiries into Maternal Deaths and Morbidity 2018-20. ," National Perinatal Epidemiology Unit, University of Oxford Oxford, 2022.

[11] B. K. Knight M, Tuffnell D, Patel R, Shakespeare J, Kotnis R, Kenyon S, Kurinczuk JJ (Eds.) on behalf of MBRRACE-UK,, "Saving Lives, Improving Mothers' Care - Lessons learned to inform maternity care from the UK and Ireland Confidential Enquiries into Maternal Deaths and Morbidity 2016-18. ," National Perinatal Epidemiology Unit, University of Oxford Oxford, 2020.

[12] O. Marshall, R. Blaylock, C. Murphy, and J. Sanders, "Risk messages relating to fertility and pregnancy: a media content analysis," *Wellcome Open Res,* vol. 6, 2021.

[13] C. M. C. Dean, "I could not survive another day. Improving treatment and tackling stigma: lessons from women's experience of abortion for severe pregnancy sickness," British Pregnancy Advisory Service, 2015.

[14] N. A. Savory, B. Hannigan, R. M. John, J. Sanders, and S. M. Garay, "Prevalence and predictors of poor mental health among pregnant women in Wales using a cross-sectional survey," *Midwifery,* vol. 103, p. 103103, 2021/12/01/ 2021, doi: https://doi.org/10.1016/j.midw.2021.103103.

For peer review only

[15] F. Stevenson *et al.*, "Decisions about the use of psychotropic medication during pregnancy: a qualitative study," *BMJ Open,* vol. 6, no. 1, p. e010130, 2016, doi: 10.1136/bmjopen-2015-010130.
[16] R. Blaylock, H. Trickey, J. Sanders, and C. Murphy, "WRISK voices: A mixed-methods study of women's experiences of pregnancy-related public health advice and risk messages in the UK," *Midwifery,* vol. 113, p. 103433, 2022/10/01/ 2022, doi: https://doi.org/10.1016/j.midw.2022.103433.
[17] Parliamentary office of Science and technology, "Infant Mortality and Stillbirth in the UK " in "POSTnote ", May 2016 2016.
[18] The NHS Race & Health Observatory. "The Power of Language: a consultation report on the use of collective terminology at the NHS Race & Health Observatory." https://www.nhsrho.org/wp-content/uploads/2021/11/NHS_RaceHealthObservatory_Terminology-consultation-report-NOV-21-1.pdf (accessed 18 August 2022).
[19] V. Braun and V. Clarke, "Using thematic analysis in psychology," *Qualitative Research in Psychology,* vol. 3, no. 2, pp. 77-101, 2006/01/01 2006, doi: 10.1191/1478088706qp063oa.
[20] M. Salmona, E. Lieber, and D. Kaczynski, *Qualitative and mixed methods data analysis using Dedoose: A practical approach for research across the social sciences*. Sage Publications, 2019.
[21] Microsoft Corporation. "Microsoft Excel." https://office.microsoft.com/excel (accessed 24 August 2022.
[22] "StataCorp. Stata Statistical Software: Release 15. College Station, TX: StataCorp LLC; 2017." (accessed.
[23] National Institute for Health and Care Excellence. "Diabetes in pregnancy: management from preconception to the postnatal period." (accessed.
[24] R. C. o. O. a. Gynaecologists, "Epilepsy In pregnancy Greentop Guideline 68," London, 2016.
[25] W. A. Shroukh, D. T. Steinke, and S. C. Willis, "Risk management of teratogenic medicines: A systematic review," (in eng), *Birth Defects Res,* vol. 112, no. 20, pp. 1755-1786, Dec 2020, doi: 10.1002/bdr2.1799.
[26] P. H. England. "BUMPS The best use of medicines in pregnancy " https://www.medicinesinpregnancy.org/Medicine--pregnancy/ (accessed 24 August 2022).
[27] J. Wise, "Pregnant women should be included in clinical trials to improve outcomes, says commission," *Bmj,* vol. 377, p. o1193, 2022, doi: 10.1136/bmj.o1193.
[28] W. G. McBride, "Thalidomide and congenital abnormalities," *The Lancet,* vol. 278, no. 7216, p. 1358, 1961/12/16/ 1961, doi: https://doi.org/10.1016/S0140-6736(61)90927-8.
[29] I. Petersen, R. L. McCrea, A. Lupattelli, and H. Nordeng, "Women's perception of risks of adverse fetal pregnancy outcomes: a large-scale multinational survey," *BMJ Open,* vol. 5, no. 6, p. e007390, 2015, doi: 10.1136/bmjopen-2014-007390.
[30] N. Vousden *et al.*, "Management and implications of severe COVID-19 in pregnancy in the UK: data from the UK Obstetric Surveillance System national cohort," *Acta Obstetricia et Gynecologica Scandinavica,* https://doi.org/10.1111/aogs.14329 vol. 101, no. 4, pp. 461-470, 2022/04/01 2022, doi: https://doi.org/10.1111/aogs.14329.

For peer review only

For peer review only

COREQ (COnsolidated criteria for REporting Qualitative research) Checklist

A checklist of items that should be included in reports of qualitative research. You must report the page number in your manuscript where you consider each of the items listed in this checklist. If you have not included this information, either revise your manuscript accordingly before submitting or note N/A.

Topic	Item No.	Guide Questions/Description	Reported on Page No.
**Domain 1: Research team and reflexivity**			
Personal characteristics			
Interviewer/facilitator	1	Which author/s conducted the interview or focus group?	
Credentials	2	What were the researcher's credentials? E.g. PhD, MD	
Occupation	3	What was their occupation at the time of the study?	
Gender	4	Was the researcher male or female?	
Experience and training	5	What experience or training did the researcher have?	
Relationship with participants			
Relationship established	6	Was a relationship established prior to study commencement?	
Participant knowledge of the interviewer	7	What did the participants know about the researcher? e.g. personal goals, reasons for doing the research	
Interviewer characteristics	8	What characteristics were reported about the inter viewer/facilitator? e.g. Bias, assumptions, reasons and interests in the research topic	
**Domain 2: Study design**			
Theoretical framework			
Methodological orientation and Theory	9	What methodological orientation was stated to underpin the study? e.g. grounded theory, discourse analysis, ethnography, phenomenology, content analysis	
Participant selection			
Sampling	10	How were participants selected? e.g. purposive, convenience, consecutive, snowball	
Method of approach	11	How were participants approached? e.g. face-to-face, telephone, mail, email	
Sample size	12	How many participants were in the study?	
Non-participation	13	How many people refused to participate or dropped out? Reasons?	
Setting			
Setting of data collection	14	Where was the data collected? e.g. home, clinic, workplace	
Presence of non-participants	15	Was anyone else present besides the participants and researchers?	
Description of sample	16	What are the important characteristics of the sample? e.g. demographic data, date	
Data collection			
Interview guide	17	Were questions, prompts, guides provided by the authors? Was it pilot tested?	
Repeat interviews	18	Were repeat inter views carried out? If yes, how many?	
Audio/visual recording	19	Did the research use audio or visual recording to collect the data?	
Field notes	20	Were field notes made during and/or after the inter view or focus group?	
Duration	21	What was the duration of the inter views or focus group?	
Data saturation	22	Was data saturation discussed?	
Transcripts returned	23	Were transcripts returned to participants for comment and/or	

For peer review only

Topic	Item No.	Guide Questions/Description	Reported on Page No.
		correction?	
**Domain 3: analysis and findings**			
Data analysis			
Number of data coders	24	How many data coders coded the data?	
Description of the coding tree	25	Did authors provide a description of the coding tree?	
Derivation of themes	26	Were themes identified in advance or derived from the data?	
Software	27	What software, if applicable, was used to manage the data?	
Participant checking	28	Did participants provide feedback on the findings?	
Reporting			
Quotations presented	29	Were participant quotations presented to illustrate the themes/findings? Was each quotation identified? e.g. participant number	
Data and findings consistent	30	Was there consistency between the data presented and the findings?	
Clarity of major themes	31	Were major themes clearly presented in the findings?	
Clarity of minor themes	32	Is there a description of diverse cases or discussion of minor themes?	

Developed from: Tong A, Sainsbury P, Craig J. Consolidated criteria for reporting qualitative research (COREQ): a 32-item checklist for interviews and focus groups. *International Journal for Quality in Health Care*. 2007. Volume 19, Number 6: pp. 349 – 357

Once you have completed this checklist, please save a copy and upload it as part of your submission. DO NOT include this checklist as part of the main manuscript document. It must be uploaded as a separate file.

For peer review only

BMJ Open

Women's experiences of over the counter and prescription medication during pregnancy in the UK: findings from survey free-text responses and narrative interviews.

Journal:	*BMJ Open*
Manuscript ID	bmjopen-2022-067987.R2
Article Type:	Original research
Date Submitted by the Author:	12-Jan-2023
Complete List of Authors:	Sanders, Julia; Cardiff University, School of Healthcare Sciences<br>Blaylock, Rebecca; British Pregnancy Advisory Service, Centre for Reproductive Research & Communication<br>Dean, Caitlin; Pregnancy Sickness Support<br>Petersen, Irene; University College London Medical School, Department of Primary Care and Population health<br>Trickey, Heather; University of Cardiff, Social Medicine<br>Murphy, Clare; British Pregnancy Advisory Service, Centre for Reproductive Research & Communication
**Primary Subject Heading**:	Obstetrics and gynaecology
Secondary Subject Heading:	General practice / Family practice, Obstetrics and gynaecology, Pharmacology and therapeutics, Qualitative research, Reproductive medicine
Keywords:	CLINICAL PHARMACOLOGY, Reproductive medicine < GYNAECOLOGY, Maternal medicine < OBSTETRICS, PUBLIC HEALTH, QUALITATIVE RESEARCH

SCHOLARONE™
Manuscripts

I, the Submitting Author has the right to grant and does grant on behalf of all authors of the Work (as defined in the below author licence), an exclusive licence and/or a non-exclusive licence for contributions from authors who are: i) UK Crown employees; ii) where BMJ has agreed a CC-BY licence shall apply, and/or iii) in accordance with the terms applicable for US Federal Government officers or employees acting as part of their official duties; on a worldwide, perpetual, irrevocable, royalty-free basis to BMJ Publishing Group Ltd ("BMJ") its licensees and where the relevant Journal is co-owned by BMJ to the co-owners of the Journal, to publish the Work in this journal and any other BMJ products and to exploit all rights, as set out in our licence.

The Submitting Author accepts and understands that any supply made under these terms is made by BMJ to the Submitting Author unless you are acting as an employee on behalf of your employer or a postgraduate student of an affiliated institution which is paying any applicable article publishing charge ("APC") for Open Access articles. Where the Submitting Author wishes to make the Work available on an Open Access basis (and intends to pay the relevant APC), the terms of reuse of such Open Access shall be governed by a Creative Commons licence – details of these licences and which Creative Commons licence will apply to this Work are set out in our licence referred to above.

Other than as permitted in any relevant BMJ Author's Self Archiving Policies, I confirm this Work has not been accepted for publication elsewhere, is not being considered for publication elsewhere and does not duplicate material already published. I confirm all authors consent to publication of this Work and authorise the granting of this licence.

For peer review only

Title

Women's experiences of over the counter and prescription medication during pregnancy in the UK: findings from survey free-text responses and narrative interviews.

Julia Sanders[a]
Rebecca Blaylock[b]
Caitlin Dean[c]
Irene Petersen[d]
Heather Trickey[e]
Clare Murphy[b]

[a] School of Healthcare Sciences, Cardiff University, United Kingdom
[b] Centre for Reproductive Research & Communication, British Pregnancy Advisory Service, 30-31 Furnival Street, London, United Kingdom
[c] Pregnancy Sickness Support, 19G Normandy Way, Bodmin, United Kingdom
[d] Department of Primary Care & Population Health, University College London, United Kingdom
[e] School of Social Sciences, Cardiff University, United Kingdom

Emails and ORCID IDs

Julia Sanders sandersj3@cardiff.ac.uk 0000-0001-5712-9989
Rebecca Blaylock rebecca.blaylock@bpas.org 0000-0003-4317-1638
Caitlin Dean caitlin@pregnancysicknesssupport.org.uk 0000-0002-8812-5101
Irene Petersen i.petersen@ucl.ac.uk 0000-0003-2446-0506
Clare Murphy clare.murphy@bpas.org 0000-0002-7272-9605

Corresponding author

Rebecca Blaylock
Research & Engagement Lead
Centre for Reproductive Research & Communication
British Pregnancy Advisory Service
30-31 Furnival Street
London
EC4A 1JQ
Email: rebecca.blaylock@bpas.org

For peer review only

References: 30

Abstract word count: 207

Word count: 4546

Abstract

Objectives: To explore women's experiences of over the counter and prescription medication advice and use during pregnancy.

Design: A study design consisting of an online survey and nested in-depth interviews with a sub-sample of participants. We analysed data from survey free-text responses and in-depth interviews using thematic analysis. Quantitative survey data is published elsewhere.

Setting: The United Kingdom.

Participants: Women were eligible if living in the UK, aged 16-45 years, were pregnant or had been pregnant in the last five years regardless of pregnancy outcome. A total of 7090 women completed the survey, and 34 women who collectively had experienced 68 pregnancies were subsequently interviewed.

Results: Medication prescribing and use during pregnancy was common. The prescribing, dispensing, and taking of some advised medications was restricted through women's or prescribers' fear of fetal harm. Lack of adherence to national prescribing guidance, conflicting professional opinion, and poor communication resulted in maternal anxiety, avoidable morbidity, and women negotiating complex and distressing pathways to obtain recommended medications. In contrast, some women felt overmedicated and that pharmacological treatments were used without exploring other options first.

Conclusions: Increased translation of national guidance into practice, and greater personalisation of antenatal care are needed to improve the safety, efficacy, and personalisation of prescribing in pregnancy.

Strengths and limitations of this study

- The study gained new insights into the seldom researched topic of medication use in pregnancy
- A sampling frame ensured inclusion of women with social and medical complexities from across the UK
- Women without sufficient spoken English or internet access were excluded
- Interviews were conducted only with women, not the health professionals who prescribe

Title

Women's experiences of over the counter and prescription medication during pregnancy in the UK: findings from survey free-text responses and narrative interviews.

Background

Safe and effective prescribing is an essential component of antenatal care. Prescribing in pregnancy requires additional knowledge and caution due to the potential for teratogenesis, altered pharmacokinetics, maternal concerns[1], and potential for short and longer term harm to the fetus[2, 3]. Antenatal information[4] provided to pregnant women states that most medications reach the fetus and all medication use should be discussed with health professionals. Many women discontinue or avoid medications in pregnancy[5], however, lack of treatment can potentially have severe consequences[1]. Despite selective avoidance, medication use in pregnancy is common with around 87% of pregnant women in the UK reporting medication use for short term or chronic conditions[6]. Excluding vitamin and iron supplements, frequently prescribed and over the counter (OTC) medications taken during pregnancy include antacids, analgesics, anti-emetics, and antibiotics[6].

There are few medications that should not be used by pregnant women, for example thalidomide, sodium valproate, and isotretinoin due to their teratogenicity. Most other medications are safe and widely used during pregnancy and there are several National

For peer review only

prescribing guidelines[7, 8] for treatment during pregnancy. For example, the National Institute for Health and Care Excellence (NICE) guidance on antenatal and postnatal mental health recommend that health professionals should discuss the potential benefits of psychological interventions and psychotropic medication, the possible consequences of no treatment and possible harms associated with treatment and what might happen if treatment is changed or stopped, particularly if psychotropic medication is stopped abruptly[8]. For mild to moderate mental health problems psychological interventions should form first line management[8], but access is limited by long waiting lists and high referral thresholds, whilst a lack of continuity in antenatal care leads to difficulty developing trusting therapeutic relationships[9]. Suicide is the leading cause of direct maternal death in the first postnatal year[10] , and reports have highlighted that treatments for depression may often be discontinued in pregnancy[5, 11].

The Royal College of Obstetricians and Gynaecologists have set out clear guidance on the diagnosis and subsequent management of nausea and vomiting in pregnancy across community, ambulatory day-care, and inpatient settings, including a medication escalation ladder for use when first line recommended treatments are ineffective[7].

There is growing concern that public health messages aimed at pregnant women, including those relating to medication use, do not always fully reflect or explain the evidence base underpinning them and the nuances and complexity of information is lost[12]. The inability to receive effective medications is not without consequence for women and babies. A survey in 2015 identified women with severe hyperemesis gravidarum frequently had difficulty obtaining swift treatment and support for debilitating pregnancy sickness resulting in some terminating an otherwise wanted pregnancy[13]. Likewise, although mental health problems are common during pregnancy[14] previous reports have highlighted barriers to access to appropriate treatment[15].

The 'WRISK project: Understanding and improving the way risk in pregnancy is communicated to women' was established to explore women's experiences of maternity related public health and risk messaging, including those relating to medication use in pregnancy. The study aimed to hear women's voices through public involvement, quantitative, and qualitative methodologies with a specific objective of exploring the views

For peer review only

of women previously identified as feeling stigmatised or poorly served by current practice[16] . This paper focuses specifically on women's experiences of medication use in pregnancy as reported in open free-text comments in the survey and in-depth interviews.

Methods

Study design

The study design consisted of an online survey and nested in-depth interviews with a sub-sample of survey participants. The survey was open to UK residents aged 16-45 years, who were pregnant or who had been pregnant in the last five years, regardless of pregnancy outcome. The survey was publicised through social media platforms including those of the study's charity partners. We invited survey respondents to express interest in further research involvement and recruited participants for the interviews from this population. As the survey was exploratory, an a priori sample size calculation was not required.

For subsequent in-depth interviews we used a sampling frame to ensure we heard the voices of women at greater risk of the most severe pregnancy outcomes, maternal death or stillbirth[11, 17]. The sampling frame aimed to ensure that 30-35 women were interviewed and included a minimum of 20% who were eligible for means-tested state benefits, a minimum of 20% from Black, Asian, and Minority Ethnic backgrounds, and at least six women were interviewed with experience of the following pregnancy experiences: pregnancy <20 years of age, Body Mass Index >30Kg/m^2, antenatal mental health problems or experience of hyperemesis gravidarum. Interested respondents who met the sampling criteria were selected using a random number table, until required numbers were obtained.

Data Collection

Survey

A participant information sheet and consent form were integrated into the survey. The survey included questions on participants' experience of the advice, information, and support they received from different sources during pregnancy, with the focus on the respondent's most recent pregnancy. Questions with open free-text responses were included alongside questions with Likert scales and multiple-choice responses. There were

For peer review only - http://bmjopen.bmj.com/site/about/guidelines.xhtml

22 questions in total, two of which asked for open free-text responses and are included in this analysis. These two questions asked:

"Please use this space to let us know about your experience and about any areas where you feel advice, information, and support for decision-making in pregnancy could be improved."

and

"Is there another issue you would like to tell us more about? If so, please tell us what this is."

We describe the survey and demographic characteristics of respondents in full in a previous publication[16]. For the purposes of this publication, we present free text responses from the survey pertaining to medication use in pregnancy only.

One-to-one interviews

The participant information sheets, consent forms, and topic guides for the interviews were developed in collaboration with the combined researcher and charity project advisory group. Narrative topic guides allowed participants to tell their pregnancy stories and were tailored to various pregnancy outcomes (see supplementary file 1). Participants were asked to primarily discuss their most recent pregnancy and were asked about antenatal prescribing and use of over-the-counter medications, amongst other topics.
Participants were offered the option of in-person or telephone interviews. Interviewers [RB and HT] made detailed field notes following each interview. Participants were offered, and all received a high street vouchers worth £20. Audio files were transcribed verbatim using a commercial transcription service. Electronic transcripts were stored separately to any identifiable data on a secure IT system, and audio files deleted once they had been checked for accuracy.

Research team

The trained, all female research team had extensive experience of working in pregnancy-related research and practice including social sciences (RB and HT), medical law (JS), public health (RB, JS, and HT) and clinical midwifery (JS). Interviews were conducted and analysed by RB and HT.

For peer review only

Terminology

The WRISK project was inclusive of all people who had experienced pregnancy in the previous five years. The project team always referred to individuals according to their self-determined gender. In this paper we use the words 'woman/ women' as the vast majority of participants self-identified as women. We use the term "BAME", but acknowledge that this is problematic[18].

Data analysis

The interviews and free text comments in the survey were analysed thematically following Braun and Clarke's method[19]. Transcripts were coded and analysed using Dedoose[20] by RB and HT. All transcripts and accompanying data such as researcher's field notes were read in detail several times by both interviewers, and high-level codes pertaining to the original research questions were identified. Further codes were identified in the data during an inductive process, resulting in a coding framework informed by the data itself. Narrative summaries of data pertaining to each code were produced and cross-checked by RB and HT, and subsequently organised into high-level themes. We used Excel® and STATA® to generate descriptive statistics on the survey and interview populations[21] [22].

Patient and Public Involvement

The project oversight group included representatives from five diverse maternity user groups, healthcare professionals, and researchers in addition to the study team. The group met regularly throughout the project from conception to dissemination and collectively informed all aspects of study design and delivery.

Results

The survey was completed by 7090 women, of whom 3,175 (44.1%) expressed willingness for further involvement in research, and 34 were subsequently interviewed. The two questions which asked for open free-text responses and included in this analysis were completed by 2,197 and 737 participants respectively. Sociodemographic characteristics of all survey respondents and quantitative survey findings are published elsewhere[16].

For peer review only

Interviews were carried out by RB and HT between July and November of 2019, lasting approximately 45-60 minutes. Two pilot interviews were conducted in-person in April 2019 and were deemed to be of sufficient quality to be included in the analysis. All subsequent interviews were conducted by telephone and recorded using a Dictaphone. The 34 women interviewed included those with experience of: a BMI >30, (n=7); antenatal medication use for mental health conditions, (n=6); medication for hyperemesis gravidarum, (n=9); age <20 during pregnancy, (n=7); or having a termination due to a perceived or actual risk either to themselves or their baby (n=7). Some participants fitted more than one category, whilst five had none of these experiences.

Sociodemographic characteristics of those interviewed are described in Table 1.Quotes that are followed by a unique identifier e.g. (WRI…) are from interview participants, and quotes that are from survey respondents are clearly identified.

Table 1

Sociodemographic characteristics of interview participants at time of interview

SOCIODEMOGRAPHICS	N (34)	%
Age		
**19-20**	2	5.9
**21-25**	9	26.5
**26-30**	7	20.6
**31-35**	7	20.6
**36-40**	7	20.6
**41-45**	0	0
**45+**	1	2.9
**Missing**	1	2.9
Highest level of education		
**Secondary school**	2	5.9
**Apprenticeship/HND/NVQ**	3	8.8
**A-Levels**	6	17.6

For peer review only

**Undergraduate degree**	13	38.2
**Postgraduate degree**	10	29.4
Relationship status		
**Married**	23	67.6
**Have a partner & live with them**	9	26.5
**Have a partner & live separately**	1	2.9
**Polyamorous**	1	2.9
Ethnicity		
**White: English/Welsh/Scottish/Northern Irish/British**	24	70.6
**Black/African/Caribbean/ Black British: African**	3	8.8
**Mixed/multiple ethnic groups: White & Black African**	1	2.9
**Asian/Asian British: Chinese**	1	2.9
**Asian/Asian British: Indian**	1	2.9
**Black/African/Caribbean/ Black British: Caribbean**	1	2.9
**Mixed/multiple ethnic groups: White & Black Caribbean**	2	5.9
**Mixed/multiple ethnic groups: White British & Middle Eastern**	1	2.9
Gender		
Female	33	97.1
Non-binary	1	2.9
Receive State benefits?		
**Yes**	10	29.4
**No**	23	67.6
**Missing**	1	2.9
**PREGNANCY HISTORY**	**Mean**	**Range**
Gravidity	2	1-5
Live births	1.2	1-4
Terminations/abortion	0.3	0-2
Miscarriage/stillbirth	0.3	0-2

Four themes were identified in relation to medication use in pregnancy: ‘fear of medications and self-regulation’, ‘feeling overmedicated’, ‘conflicting opinions’, and ‘running the gauntlet’. Some themes related to women’s experiences of medication use in general, while others were condition specific.

Fear of medications and self-regulation

Fear, or anxiety for the potential for fetal harm caused by over the counter and prescribed medications were expressed by women. They also reported prescribers’ reluctance to

For peer review only - http://bmjopen.bmj.com/site/about/guidelines.xhtml

prescribe for this reason. Participants expressed support for the precautionary approach that medications should be kept to a minimum during pregnancy. The fear of causing fetal harm through taking medication resulted in women reducing medication even when this was required to control serious medical conditions.

Two women described the consequences of stopping prescribed medication for pre-existing medical conditions. One woman stopped taking her asthma medication which resulted in hospitalisation:

"I stopped taking my inhaler, just because I just didn't want to harm the baby. I took a bad cold, and I was coughing up blood, and then they sent me to hospital for all these other tests. They thought I had a clot or something. So that obviously scared me. Then I just said, Screw it. I'm just going to take my asthma medication, and that was that." [WRI28]

Similarly, another woman experienced a serious migraine and temporary blindness, following discontinuation of medication for cranial hypertension:

"I have a brain condition called... Well, it's cranial hypertension, so it just means that my body produces too much spinal fluid, which pushes on my brain, which causes migraines, essentially. … because there isn't a lot of research into this condition in pregnancy, I decided, personally, to stop taking the medication. Yes, so I stopped all my medication and, thankfully, touch wood, everything was fine. … I did struggle with a few migraines throughout the pregnancy, one which was quite severe, which resulted in a loss of vision in my left eye, but luckily, it came back. So that was fine. That was quite difficult to deal with, though." [WRI23]

Self-regulation of medication particularly related to the use of analgesics. Women described conflict between their understanding of some medications being safe to take in pregnancy, with their desire to try "not to take them" [WRI11] due to the possibility of potential risks to the fetus.

For peer review only

"… we all know it's [paracetamol] is generally safe, it's one of the safest painkillers there is, so it's the only one you can take, but even just taking that, women don't like- you know, they have a headache instead of taking the medicine, because they feel so guilty." [WRI30]

Another woman described managing with paracetamol for pelvic girdle pain even after she had been prescribed codeine-based medication:

"Yes, one of those [codeine-based analgesia] and I was reading the side effects and I was like, 'No, I'm not happy taking it and having that go into my baby.' I just literally was taking paracetamol and I was just trying to take the lowest dose of paracetamol that I could really." [WRI4]

Whilst women generally tried to avoid prescribed medication, recommended vaccines, including for influenza, appeared to be accepted. Supplements were viewed as positive and often taken without any apparent fear of causing an adverse pregnancy outcome.

"Interviewer: Did you take medications while you were pregnant?

No, not at all.

Interviewer: No, not at all. Okay.

Beyond supplements and the flu jab and recommended stuff like that. I had a whooping cough jab, I think, with [daughter's name] but no regular medication of any sort." (WRI7)

There were many examples of health professionals reinforcing the message that medication should be avoided in pregnancy.

"Throughout my pregnancy I really struggled with my mental health. So, at one point, I don't know how pregnant I was, I must have been about six or seven months, we got an emergency appointment at my GP's surgery… and we said, 'I'm really struggling. Is there anything you can do?' His first reaction was that I shouldn't be on any medication because I was pregnant." [WRI29]

For peer review only

Health professionals expressing their own concerns around prescribing had a particularly negative impact on women. One interviewee [WRI17] explained her concern when a hospital consultant prescribing "put his hands together in prayer and said, '*God, forgive me for giving you this. I hope your baby's okay*'" when prescribing ondansetron for severe hyperemesis gravidarum (HG).

Other women had similar experiences when accessing medication for their mental health. WRI33 said:

> *"because I was being so sick, I went to the doctor's about anti-sickness medication. She saw I was on antidepressants and she made me feel like I was the world's worst mother. I don't know whether she meant to, but she was just worried that, 'Ooh, you're pregnant and you're on medication.' She made me feel like, 'You're hurting your baby'. Then when I went to the pharmacy to pick up my meds, they made me feel like it on the same day as well. I think that was the point where I started to really decline in my mental health. It just seemed to trigger something off that I was going to be a terrible mother." [WRI33]*

Feeling overmedicated

Some participants reported feeling overmedicated, and that pharmacological treatments were used without exploring other options first. This was the case for women with both mental and physical health concerns:

> *"I feel like they push drugs on people too quickly, that is just the go-to. I had a traumatic birth with number two. That's why I went on the antidepressants with number three, because I was terrified of giving birth. They just kept telling me baby was getting so big, blah blah blah. I was, literally, terrified of giving birth to her. They just put my antidepressants. There was no, 'Let's talk about it, get counselling, and get over the traumatic birth.' It was just mask it really. I don't think that's right to do when you're dealing with, obviously, a pregnant woman, a baby. [WRI31]*

Current guidance relating to gestational diabetes recommends metformin is introduced if good blood glucose control is not achieved with 1 – 2 weeks of dietary change and

For peer review only - http://bmjopen.bmj.com/site/about/guidelines.xhtml

exercise[23]. One survey respondent would have preferred to extend this duration before commencing medication.

“I would have liked to have been better involved in decision making around my options for treatment in relation to my gestational diabetes… I ended up with 2 tablets of metformin which I think was overkill - I would have liked to have had the opportunity to try harder with diet and exercise before starting medication.” [Survey respondent]

Conflicting opinions

Women who required prescribed medication in pregnancy described frustration and distress resulting from conflicting opinions of health professionals. This conflict was particularly felt by women with hyperemesis gravidarum or mental health problems. One woman who required ondansetron for severe hyperemesis gravidarum was prescribed it, later to have this decision questioned by another General Practitioner [GP] in the same practice:

“So, I was prescribed by one doctor the ondansetron and because I was running out, they said I had to speak to another doctor just to check it was obviously working, just a check-up on it, to get the repeat prescription. Then when this doctor phoned me, she said, ‘You shouldn’t be taking this. This is for cancer patients. There’s no reason why you should be taking it. You’re putting your baby’s life at risk’. … She just wasn’t having any of it. I even told her that I’d just had a scan like 45 minutes before the phone call, and she said, ‘Oh well, that can change at your 20 week scan. Don’t come crying to me when they tell you that your baby has got heart defects’”. [WRI15]

Another participant described the distress that resulted when a pharmacist would not fulfil a prescription for anti-depressants prescribed by her GP.

“They just wouldn't give me my prescription, and people were behind me in the queue and could hear what I was on, that I was pregnant. I wasn't 12

For peer review only

weeks yet, so I hadn't told anybody. I had to just sit there waiting for them to decide whether I could have it or not and then they said, 'No, you can't have it.' I left without any medication, and I was just crying in the car." [WRI33]

Two interview participants said that conflicting and contradictory advice from healthcare professionals was "staggering".

"I found through everything in my pregnancy that all the doctors I saw had completely different advice and completely different opinions to each other..." [WRI6]

The conflict was seen across all professional groups. One respondent described conflicting information from her midwife and GP.

"I was advised by my GP to continue my anti-depressant medication. My midwife continually questioned this decision throughout the whole pregnancy". [survey respondent]

Running the Gauntlet

This theme particularly related to prescribing for hyperemesis gravidarum. Some women experienced prolonged periods prior to getting effective treatment. Four women who were suffering with hyperemesis gravidarum described their experience of the current guidance.

"they tried me on cyclizine, which just made me dizzy and did nothing for the sickness. I went very dizzy and sleepy. And then Stemetil… Which also had no effect. So, I was, kind of, getting desperate. And then, … they gave me ondansetron which is what they give to chemo patients, you probably know, and it was like I was alive again. … They were sympathetic, but I didn't get a lot of advice on what effect it would have on the baby. Just, you know, like, with the Cyclzine and the Stemetil, they were very much, like, 'It's been used a lot and we can be certain from observations that it won't have any effect.' So that was fine. They were, kind of, reluctant to, like, move me onto the stronger stuff." [WRI1]

For peer review only

*"… they could see that I couldn't even take a sip of water without being sick, so I needed something. So, first off, I was given a tablet that you put between your gum and your teeth. It wasn't working. So, I went back and I got something called cyclizine, which is a tablet. That started to make it a little bit better for a couple of weeks and then I just got worse again. So, I had to go back again and …[they] gave me a pill called ondansetron. Then that was working, but I was still quite bad. So, I went back again and they gave me a combination of cyclizine and ondansetron, and that did the trick in the end". **[**WRI6]*

Even when women were prescribed treatment for hyperemesis, they could then be discouraged from taking it by the prescriber:

"The most unhelpful piece of advice I received was from a doctor, who prescribed me anti-sickness tablets for hyperemesis but said 'try not to take them unless you feel you need them' because 'we can't say that any medicine is safe in pregnancy'. I was very unwell, very dehydrated and very disoriented and was afraid of causing harm to the baby so I didn't take them." [Survey respondent]

Another woman who was prescribed medication received comments from a pharmacist who suggested they may be unnecessary:

"My husband went to collect my prescription when I was discharged, and was told that I was being 'irresponsible' and 'putting the baby at risk' by taking the medication. Later in pregnancy (I had HG until the moment of delivery), another pharmacist told me that I shouldn't still be taking medication as morning sickness would've stopped by my late stage, implied it was all in my head, and suggested that I try a herbal remedy instead." [Survey respondent]

For peer review only

Discussion

This study adds important understanding to the seldom explored topic of women's experiences of antenatal medication use. Many women wished to reduce exposure to over the counter and prescribed medication use during pregnancy, but dietary supplements and vaccines, were generally accepted by pregnant women. This contrasted to treatments for mental health problems and hyperemesis gravidarum, conditions where pregnant women described having to negotiate conflicting opinions of health professionals to obtain recommended or effective treatments. Too often prescribing was more restrictive than recommended in national guidance resulting in avoidable, or prolonged, maternal morbidity, distress, and anxiety. Similar to the lack of preconceptual and antenatal care for women with epilepsy highlighted in reviews of maternal deaths[11], where information on medications used for chronic conditions was not shared, some women discontinued treatments without medical consultation, resulting in hospitalisation or exacerbation of symptoms.

In the UK, prescribing for pregnant women is undertaken by different health professionals which complicates communication. Women with existing medical conditions, including epilepsy, require preconceptual advice on medication use[24]. GPs are frequently involved in prescribing decisions, commonly prescribed medications such as iron therapy and low dose aspirin may be prescribed and administered by midwives. Women with additional obstetric needs will receive care led by obstetricians, who may share prescribing with GPs or specialist physicians. This arena is further complicated by public health bodies who are responsible for producing public health risk messages for pregnancy being independent from both primary and secondary care where prescribing occurs. This multidisciplinary approach to antenatal prescribing was found often to be fragmented, with women hearing conflicting opinions even from different members of the same professional group. The role of pharmacists as medication 'gatekeepers' and their refusal to dispense prescribed medications highlights the importance of their inclusion in system improvements.

Even when effective treatments were endorsed in national guidance, challenges in implementation were found. Despite RCOG guidance on the management of hyperemesis gravidarum[7], women reported being denied access to effective treatments. Ensuring

For peer review only

health professionals have easy access to up-to-date guidance on specialist aspects of care, and sufficient time and support to incorporate guidance into their practice is imperative. Whilst prescribers need to balance maternal benefit with potential fetal harm when prescribing in pregnancy, women's individual circumstances were not always considered, and they were not fully engaged in decision making. Possibly reflecting the tendency of health professionals to overestimate the teratogenic potential of drugs[25] we found many examples where health professionals used fear of fetal harm to justify a refusal to prescribe or dispense otherwise recommended medications. This had a significant impact not only on women's health, but on their emotional wellbeing with one reporting they were made to feel like the "world's worst mother".

Some women felt antidepressants and metformin for gestational diabetes were offered in preference to non-pharmacological options. This may reflect that the availability of talking therapies does not meet demand, or in relation to gestational diabetes, a lack of informed personalised conversations on the effectiveness of methods to obtain glycaemic control.

High quality, easily accessible information on the safety of medicines is available[26] but appeared to be underutilised in informing individualised discussions. Better reporting systems on outcomes related to medication use in pregnancy are needed, including making the results of clinical trials more generalisable through, where appropriate, the inclusion of pregnant women[27]. Minimising prescribing in pregnancy became deeply entrenched in the ethos of antenatal care following the thalidomide tragedy[28]. The belief that all medication use during pregnancy carries risk is commonly held among women, with paracetamol, antibiotics and antidepressants[29] considered to be on a continuum of increasing risk. The potential for this position to cause harm is increasingly being realised. Some women with epilepsy, and others with serious mental health conditions have died because of an over stringent position on medication avoidance[11]. More recently the numbers of pregnant women who died during the covid-19 pandemic could have been reduced through earlier acceptance of the safety of vaccination in pregnancy, better public health communication, and greater use of effective treatments among those seriously ill with COVID-19[30]. While the need to reduce maternal mortalities through improved prescribing is already appreciated, our study suggests physical and mental morbidity caused

For peer review only

through the lack of access to effective treatments for mental health conditions and hyperemesis gravidarum is likely to be very common and requires improvement.

Strengths of this study include adding to knowledge on a seldom explored topic, the geographical spread and high number of survey participants from across the UK, and use of a sampling frame for the selection of interview participants. A weakness was that the sampling frame design may have shaped our findings and focused attention on certain experiences of medication use at the expense of others of equal concern to women. Our survey was self-selecting and may reflect the views of those more motivated to participate in research. The survey was only available in English and via the internet, excluding some groups from participation including non-English reading women, and those with limited or no access to the internet. This study would have been strengthened by the inclusion of clinicians to better understand their attitudes and understandings of prescribing and dispensing during pregnancy.

Conclusion

Medication prescribing and use during pregnancy is common. However, fear of fetal harm restricts the prescribing and taking of some advised medications. Analgesics were commonly avoided or taken at lower than the therapeutic or prescribed dose and some women reduced or discontinued medications for chronic conditions without medical oversight. Where existing clinical guidance was not followed, or there was conflict in professional opinion, reluctance to prescribe or dispense resulted in women needing to negotiate complex and distressing pathways to obtain required medications. The study identified aspects of antenatal prescribing where improvement in knowledge, communication, or practice is required to ensure maximisation of the safety, efficacy, and personalisation of prescribing in pregnancy.

For peer review only

Funding statement

This work was supported by the Wellcome Trust as part of the WRISK Project [212089/Z/18/Z].

Data availability statement

Data are available on request.

Ethics statements

Patient consent for publication

Not applicable.

Ethical approval

Ethical approval was granted by the Research and Ethics committee of the School of Social Sciences at Cardiff University SREC/3201.

Competing interests

None

Author Contributions

CM and HT conceptualised the study and secured funding. CM, RB, HT, JS, IP and CD developed the protocol. HT and RB conducted the data collection and analysis. CM, JS, HT and RB provided study management. JS drafted the original manuscript. RB, IP and CD contributed to writing. CD and IP were members of the project advisory group. All authors approved the final manuscript.

Acknowledgements

We acknowledge and thank the group of over 7,000 women who shared their intimate stories of pregnancy with us. Special thanks go to the Oversight Committee: Dame Cathy Warwick, BPAS Chair of Board of Trustees and Chair of the Oversight Committee; Rebecca Brione, Birthrights; Jane Fisher, Antenatal Results and Choices; Caitlin Dean, Pregnancy Sickness Support; Elizabeth Duff, NCT; Professor Irene Petersen, University College London; Amber Marshall, BigBirthas.co.uk; Professor Fiona Woollard, Southampton University; Rolling representative, Public Health Wales.
We dedicate this paper to the late Dr Heather Trickey, without whom the project would never exist. Thank you for your inspiration, and dedication to the health and wellbeing of all women.

For peer review only

For peer review only

References

[1] M. J. Twigg, A. Lupattelli, and H. Nordeng, "Women's beliefs about medication use during their pregnancy: a UK perspective," (in eng), *Int J Clin Pharm,* vol. 38, no. 4, pp. 968-976, 2016, doi: 10.1007/s11096-016-0322-5.

[2] W. H. Meeraus, I. Petersen, and R. Gilbert, "Association between antibiotic prescribing in pregnancy and cerebral palsy or epilepsy in children born at term: a cohort study using the health improvement network," *PLoS ONE [Electronic Resource],* Research Support, Non-U.S. Gov't vol. 10, no. 3, p. e0122034, 2015. [Online].

[3] L. Page, "Prescribing for pregnancy: managing prescribing for women with mental health diagnoses," *Drug & Therapeutics Bulletin,* Review vol. 58, no. 1, pp. 8-11, 2020. [Online].

[4] N. England. "Medicines in pregnancy." https://www.nhs.uk/pregnancy/keeping-well/medicines/ (accessed 24 August 2022.

[5] I. Petersen, R. E. Gilbert, S. J. Evans, S. L. Man, and I. Nazareth, "Pregnancy as a major determinant for discontinuation of antidepressants: an analysis of data from The Health Improvement Network," (in eng), *J Clin Psychiatry,* vol. 72, no. 7, pp. 979-85, Jul 2011, doi: 10.4088/JCP.10m06090blu.

[6] A. Lupattelli *et al.*, "Medication use in pregnancy: a cross-sectional, multinational web-based study," *BMJ Open,* vol. 4, no. 2, p. e004365, 2014, doi: 10.1136/bmjopen-2013-004365.

[7] Royal College of Obstetricians and Gynaecologists, "The Management of Nausea and Vomiting of Pregnancy and Hyperemesis Gravidarum," in "Green-top Guideline No. 69," London, 2016.

[8] National Insitute for Health and Care Excellence, "Antenatal and postnatal mental health: clinical management and service guidance. Clinical Guideline 192," 2020. [Online]. Available: www.nice.org.uk/guidance/cg192

[9] O. Megnin-Viggars, I. Symington, L. M. Howard, and S. Pilling, "Experience of care for mental health problems in the antenatal or postnatal period for women in the UK: a systematic review and meta-synthesis of qualitative research," (in eng), *Arch Womens Ment Health,* vol. 18, no. 6, pp. 745-59, Dec 2015, doi: 10.1007/s00737-015-0548-6.

[10] B. K. Knight M, Tuffnell D, Patel R, Shakespeare J, Kotnis R, Kenyon S, Kurinczuk JJ (Eds.) on behalf of MBRRACE-UK,, "Saving Lives, Improving Mothers' Care - Lessons learned to inform maternity care from the UK and Ireland Confidential Enquiries into Maternal Deaths and Morbidity 2018-20. ," National Perinatal Epidemiology Unit, University of Oxford Oxford, 2022.

[11] B. K. Knight M, Tuffnell D, Patel R, Shakespeare J, Kotnis R, Kenyon S, Kurinczuk JJ (Eds.) on behalf of MBRRACE-UK,, "Saving Lives, Improving Mothers' Care - Lessons learned to inform maternity care from the UK and Ireland Confidential Enquiries into Maternal Deaths and Morbidity 2016-18. ," National Perinatal Epidemiology Unit, University of Oxford Oxford, 2020.

[12] O. Marshall, R. Blaylock, C. Murphy, and J. Sanders, "Risk messages relating to fertility and pregnancy: a media content analysis," *Wellcome Open Res,* vol. 6, 2021.

[13] C. M. C. Dean, "I could not survive another day. Improving treatment and tackling stigma: lessons from women's experience of abortion for severe pregnancy sickness," British Pregnancy Advisory Service, 2015.

[14] N. A. Savory, B. Hannigan, R. M. John, J. Sanders, and S. M. Garay, "Prevalence and predictors of poor mental health among pregnant women in Wales using a cross-sectional survey," *Midwifery,* vol. 103, p. 103103, 2021/12/01/ 2021, doi: https://doi.org/10.1016/j.midw.2021.103103.

[15] F. Stevenson *et al.*, "Decisions about the use of psychotropic medication during pregnancy: a qualitative study," *BMJ Open,* vol. 6, no. 1, p. e010130, 2016, doi: 10.1136/bmjopen-2015-010130.

[16] R. Blaylock, H. Trickey, J. Sanders, and C. Murphy, "WRISK voices: A mixed-methods study of women's experiences of pregnancy-related public health advice and risk messages in the UK," *Midwifery,* vol. 113, p. 103433, 2022/10/01/ 2022, doi: https://doi.org/10.1016/j.midw.2022.103433.

[17] Parliamentary office of Science and technology, "Infant Mortality and Stillbirth in the UK " in "POSTnote ", May 2016 2016.

[18] The NHS Race & Health Observatory. "The Power of Language: a consultation report on the use of collective terminology at the NHS Race & Health Observatory." https://www.nhsrho.org/wp-content/uploads/2021/11/NHS_RaceHealthObservatory_Terminology-consultation-report-NOV-21-1.pdf (accessed 18 August 2022).

[19] V. Braun and V. Clarke, "Using thematic analysis in psychology," *Qualitative Research in Psychology,* vol. 3, no. 2, pp. 77-101, 2006/01/01 2006, doi: 10.1191/1478088706qp063oa.

[20] M. Salmona, E. Lieber, and D. Kaczynski, *Qualitative and mixed methods data analysis using Dedoose: A practical approach for research across the social sciences*. Sage Publications, 2019.

[21] Microsoft Corporation. "Microsoft Excel." https://office.microsoft.com/excel (accessed 24 August 2022.

[22] "StataCorp. Stata Statistical Software: Release 15. College Station, TX: StataCorp LLC; 2017." (accessed.

[23] National Institute for Health and Care Excellence. "Diabetes in pregnancy: management from preconception to the postnatal period." (accessed.

[24] R. C. o. O. a. Gynaecologists, "Epilepsy In pregnancy Greentop Guideline 68," London, 2016.

[25] W. A. Shroukh, D. T. Steinke, and S. C. Willis, "Risk management of teratogenic medicines: A systematic review," (in eng), *Birth Defects Res,* vol. 112, no. 20, pp. 1755-1786, Dec 2020, doi: 10.1002/bdr2.1799.

[26] P. H. England. "BUMPS The best use of medicines in pregnancy " https://www.medicinesinpregnancy.org/Medicine--pregnancy/ (accessed 24 August 2022).

[27] J. Wise, "Pregnant women should be included in clinical trials to improve outcomes, says commission," *Bmj,* vol. 377, p. o1193, 2022, doi: 10.1136/bmj.o1193.

[28] W. G. McBride, "Thalidomide and congenital abnormalities," *The Lancet,* vol. 278, no. 7216, p. 1358, 1961/12/16/ 1961, doi: https://doi.org/10.1016/S0140-6736(61)90927-8.

[29] I. Petersen, R. L. McCrea, A. Lupattelli, and H. Nordeng, "Women's perception of risks of adverse fetal pregnancy outcomes: a large-scale multinational survey," *BMJ Open,* vol. 5, no. 6, p. e007390, 2015, doi: 10.1136/bmjopen-2014-007390.

[30] N. Vousden *et al.*, "Management and implications of severe COVID-19 in pregnancy in the UK: data from the UK Obstetric Surveillance System national cohort," *Acta Obstetricia et Gynecologica Scandinavica,* https://doi.org/10.1111/aogs.14329 vol. 101, no. 4, pp. 461-470, 2022/04/01 2022, doi: https://doi.org/10.1111/aogs.14329.

For peer review only

For peer review only

WRISK Topic Guide

Consent

Thank you … I'm part of a small research team run by the British Pregnancy Advisory Service and researchers at Cardiff University. Today I'd like to explore your thoughts, opinions, and experiences of information and advice you received about your last pregnancy.

Check: are you still happy to be involved with the research?

Just to check, I will be recording the interview… **(If face-to-face, show participant the audio-recorder).** It will help me to remember what we talk about and means I do not have to make loads of notes whilst we are talking. The tape will not be played to anyone outside of the research team.

Are you happy with that?

Read written consent form with participant, checking;

- **understand the statement,**
- **agree with it, and,**
- **have had opportunity to ask questions and that boxes are all checked.**

Our conversation will remain confidential.
You will not be named in anything I write about this research.

If there is anything you don't want to talk about just say so- some questions may involve recalling distressing experiences. This interview is about your experiences. If we start to discuss something that you would rather not, please let me know and we can talk about something else.

These interviews usually last about one hour, but we can stop whenever you like. If you would like to take a break, please just let me know.

About you and your most recent pregnancy

Perhaps we can start with you and your family … can you tell me a little bit about that …

Allow participant to talk, In course of which ask…

- **Number and ages of any children …**
- **Most recent pregnancy…**
- **Other pregnancies, miscarriages, terminations …**
  (but take a judgement about not interrupting flow)

Okay, so thinking a little bit about your most recent pregnancy ….
Can you tell me a little about that?

- What would you say were the highpoints- Why?
- What would you say were the low points - Why?

For peer review only - http://bmjopen.bmj.com/site/about/guidelines.xhtml

Remaining topics can be covered in any order.

In wider life context

Women often have a lot going on in their lives during the time when they are pregnant … for some people it can be a time of when everything seems to happen at once…

What else was going on for you at that time?

Explore – relationships and partners, housing, work, education …

Becoming pregnant

So… would you say this pregnancy was …

- Planned, or sort-of planned, or a surprise …

Thinking back to the months before became pregnant …
Did you have any concerns about…

- **Living circumstances, work, family life**
- **Fertility or about being pregnant**
- **Your own health**

Thinking back, do you remember getting any information or advice about looking after yourself before becoming pregnant? What? And Who?

- **Health professionals**
- **Books / social media**
- **Friends and family**

Did you seek out this advice yourself?

Did you get any help that you felt was particularly helpful? Unhelpful?

Was there anything you wish someone had told you then?

Finding out

Now, thinking back to when you found out that you were pregnant…
Can you tell me a little bit about that?

Prompt

- **How did you feel about finding out that you were pregnant?**
- **Did you have any particular concerns?**
  - **Health**
  - **Work, housing, social life**
  - **Life circumstances/partners/support**

For peer review only - http://bmjopen.bmj.com/site/about/guidelines.xhtml

Did anyone advise or tell you anything that you felt was particularly helpful / unhelpful at that time? Is there anything you would like to have known?

What? And Who?

Did you look for any information/advice yourself? For example on the internet, in books, or magazines?

Stories in the media

There are often lots of stories about health and pregnancy on social media, newspapers, magazines, tv and online, talking about things mothers should and shouldn't do to conceive or have a healthy pregnancy and baby. Have you been aware of any stories like that?

Prompt – for example, about what to eat or drink, or activities not to take part in?
Prompt – what did you think about those stories?
Prompt- how did they make you feel, if anything?
Prompt – did any stories feel particularly relevant to you?
Prompt – did you make any changes because of the stories you had read?

Health and medications

Many women take medication while they are pregnant – did you take any medications?

If yes… which ones? When?

Did you get any information or advice from health professionals, friends, family members or from social and mainstream media about taking medication?

If yes… explore, If no… why not… explore

How did you feel about the advice or information that you got?

Did you feel that advice from different people was broadly similar or not?
Was there anything that you felt was particularly helpful? / Particularly unhelpful?
Did you decide to do anything differently?

Lifestyle advice

Sometimes women are given specific information about things like eating and drinking in pregnancy from health professionals or family members or friends or from social media or mainstream media … do you remember receiving any information or advice about…

- **Healthy lifestyle**
- **Weight and exercise**
- **Alcohol**
- **Smoking**
- **Cannabis, Cocaine, Ecstasy**

What advice/ information? And who gave it?

Was there anything that you felt was particularly helpful? / Particularly unhelpful?

For peer review only - http://bmjopen.bmj.com/site/about/guidelines.xhtml

Did you decide to do anything differently? Why? Did you change the way you did things because you wanted to or because you felt under pressure to?

Weight and fitness

Sometimes women are given information or advice about managing their weight and general fitness.

Did you get any advice and information or information about managing your weight or level of fitness?

If yes… explore,

Can you tell me about any information or advice you were given?
How did you feel about that advice?

Was there anything that you felt was particularly helpful? / Particularly unhelpful?
Did you decide to do anything differently?

Antenatal appointments and scans

I would like to hear about your experience of going for antenatal care and scans…

Can you tell me about your experience of going for a scan?

- What info or advice did you have? Helpful? Not?

What advice did you receive about antenatal care? And who gave it? (Probe third sector orgs etc. if appropriate)

Was there anything that you felt was particularly helpful? / Particularly unhelpful?

Did you decide to do anything differently?

Planning for birth

Thinking now about the birth of your baby…. Can you tell me a little about that?

Did things go the way you hoped they would?

Explore how decisions were made and how mother felt about those decisions… including exploring any sources of risk advice – health professional (specifically which one?), friend or family member, social media, antenatal teacher etc… associated with ….

- **Type of delivery**
- **Assisted?**
- **Place of birth**
- **Timing of delivery/induction**
- **Probe: quantitative presentation of risk information**

Do you think the information you were given helped you make the decision that was right for you?
Did you feel listened to and respected?

Looking back

For peer review only - http://bmjopen.bmj.com/site/about/guidelines.xhtml

Looking back along the whole of your pregnancy, if you could go back in time and do something differently in the time before you became pregnant … what would that be? Why?

[IF APPLICABLE: Anything different compared to previous pregnancies?]

And again, thinking back, is there anything you wished you had or hadn't known?

If you could change anything about the advice and support that you received during pregnancy and birth, what would it be …

Final thoughts and thank you

We have almost come to the end of our time together. Is there anything else you would like to add to our conversation today?

Thank you so much for your time today. Everything you have shared today has been so helpful.

Thank participant and give (or remind that you are emailing) a £20 gift voucher as a thank you.

I'm going to turn off the recorder now.

I am going to go away and transcribe what I have recorded today. You can contact me at any time to discuss what we have shared or if you have any questions about the research.

For peer review only - http://bmjopen.bmj.com/site/about/guidelines.xhtml

COREQ (COnsolidated criteria for REporting Qualitative research) Checklist

A checklist of items that should be included in reports of qualitative research. You must report the page number in your manuscript where you consider each of the items listed in this checklist. If you have not included this information, either revise your manuscript accordingly before submitting or note N/A.

Topic	Item No.	Guide Questions/Description	Reported on Page No.
**Domain 1: Research team and reflexivity**			
Personal characteristics			
Interviewer/facilitator	1	Which author/s conducted the interview or focus group?	
Credentials	2	What were the researcher's credentials? E.g. PhD, MD	
Occupation	3	What was their occupation at the time of the study?	
Gender	4	Was the researcher male or female?	
Experience and training	5	What experience or training did the researcher have?	
Relationship with participants			
Relationship established	6	Was a relationship established prior to study commencement?	
Participant knowledge of the interviewer	7	What did the participants know about the researcher? e.g. personal goals, reasons for doing the research	
Interviewer characteristics	8	What characteristics were reported about the inter viewer/facilitator? e.g. Bias, assumptions, reasons and interests in the research topic	
**Domain 2: Study design**			
Theoretical framework			
Methodological orientation and Theory	9	What methodological orientation was stated to underpin the study? e.g. grounded theory, discourse analysis, ethnography, phenomenology, content analysis	
Participant selection			
Sampling	10	How were participants selected? e.g. purposive, convenience, consecutive, snowball	
Method of approach	11	How were participants approached? e.g. face-to-face, telephone, mail, email	
Sample size	12	How many participants were in the study?	
Non-participation	13	How many people refused to participate or dropped out? Reasons?	
Setting			
Setting of data collection	14	Where was the data collected? e.g. home, clinic, workplace	
Presence of non-participants	15	Was anyone else present besides the participants and researchers?	
Description of sample	16	What are the important characteristics of the sample? e.g. demographic data, date	
Data collection			
Interview guide	17	Were questions, prompts, guides provided by the authors? Was it pilot tested?	
Repeat interviews	18	Were repeat inter views carried out? If yes, how many?	
Audio/visual recording	19	Did the research use audio or visual recording to collect the data?	
Field notes	20	Were field notes made during and/or after the inter view or focus group?	
Duration	21	What was the duration of the inter views or focus group?	
Data saturation	22	Was data saturation discussed?	
Transcripts returned	23	Were transcripts returned to participants for comment and/or	

For peer review only

Topic	Item No.	Guide Questions/Description	Reported on Page No.
		correction?	
**Domain 3: analysis and findings**			
Data analysis			
Number of data coders	24	How many data coders coded the data?	
Description of the coding tree	25	Did authors provide a description of the coding tree?	
Derivation of themes	26	Were themes identified in advance or derived from the data?	
Software	27	What software, if applicable, was used to manage the data?	
Participant checking	28	Did participants provide feedback on the findings?	
Reporting			
Quotations presented	29	Were participant quotations presented to illustrate the themes/findings? Was each quotation identified? e.g. participant number	
Data and findings consistent	30	Was there consistency between the data presented and the findings?	
Clarity of major themes	31	Were major themes clearly presented in the findings?	
Clarity of minor themes	32	Is there a description of diverse cases or discussion of minor themes?	

Developed from: Tong A, Sainsbury P, Craig J. Consolidated criteria for reporting qualitative research (COREQ): a 32-item checklist for interviews and focus groups. *International Journal for Quality in Health Care*. 2007. Volume 19, Number 6: pp. 349 – 357

Once you have completed this checklist, please save a copy and upload it as part of your submission. DO NOT include this checklist as part of the main manuscript document. It must be uploaded as a separate file.

For peer review only
